# Genomics of Rapid Adaptation to Antibiotics: Convergent Evolution and Scalable Sequence Amplification

**DOI:** 10.1093/gbe/evu106

**Published:** 2014-05-20

**Authors:** David Laehnemann, Rafael Peña-Miller, Philip Rosenstiel, Robert Beardmore, Gunther Jansen, Hinrich Schulenburg

**Affiliations:** ^1^Department of Evolutionary Ecology and Genetics, University of Kiel, Germany; ^2^Biosciences, Geoffrey Pope Building, University of Exeter, United Kingdom; ^3^Department of Zoology, University of Oxford, United Kingdom; ^4^Institute for Clinical Molecular Biology, University of Kiel, Germany

**Keywords:** antibiotic resistance, *Escherichia coli*, experimental evolution, MC4100, synonymous codon, population genomics

## Abstract

Evolutionary adaptation can be extremely fast, especially in response to high selection intensities. A prime example is the surge of antibiotic resistance in bacteria. The genomic underpinnings of such rapid changes may provide information on the genetic processes that enhance fast responses and the particular trait functions under selection. Here, we use experimentally evolved *Escherichia coli* for a detailed dissection of the genomics of rapid antibiotic resistance evolution. Our new analyses demonstrate that amplification of a sequence region containing several known antibiotic resistance genes represents a fast genomic response mechanism under high antibiotic stress, here exerted by drug combination. In particular, higher dosage of such antibiotic combinations coincided with higher copy number of the sequence region. The amplification appears to be evolutionarily costly, because amplification levels rapidly dropped after removal of the drugs. Our results suggest that amplification is a scalable process, as copy number rapidly changes in response to the selective pressure encountered. Moreover, repeated patterns of convergent evolution were found across the experimentally evolved bacterial populations, including those with lower antibiotic selection intensities. Intriguingly, convergent evolution was identified on different organizational levels, ranging from the above sequence amplification, high variant frequencies in specific genes, prevalence of individual nonsynonymous mutations to the unusual repeated occurrence of a particular synonymous mutation in Glycine codons. We conclude that constrained evolutionary trajectories underlie rapid adaptation to antibiotics. Of the identified genomic changes, sequence amplification seems to represent the most potent, albeit costly genomic response mechanism to high antibiotic stress.

## Introduction

Bacterial populations are able to adapt to environmental stress extremely rapidly. This has best been documented with the help of evolution experiments in the laboratory, for which environmental conditions can be precisely controlled, permitting reliable inference of cause–effect relationships (Kawecki et al. 2012). One of the classic examples is the adaptation of *Escherichia coli* to one of its phages in less than 5 days (Lenski and Levin 1985). More recent examples refer to a 5,000-fold increase in resistance of *E. coli* to strong ionizing radiation within 20 selection cycles (Harris et al. 2009) or the substantially increased fitness of *E. coli* in extreme temperature environments within 2,000 generations (Tenaillon et al. 2012). Perhaps the most compelling evidence for swift bacterial adaptation comes from work on antibiotic resistance evolution. Within merely 2 days after onset of drug deployment, experimental *E. coli* populations restore growth to almost untreated levels (Hegreness et al. 2008). Such fast antibiotic resistance evolution represents a global health problem (Palumbi 2001; Jacoby 2009), and although comprehensive information is available on the molecular basis of resistance (Walsh 2000, 2003; Alekshun and Levy 2007), the mechanisms, patterns, and processes underlying its evolution are still only poorly understood (MacLean et al. 2010).

One particular challenge of current research therefore is to understand the genomic underpinnings of such fast adaptive changes. We here assume that adaptation is based on evolution (i.e., a change in allele frequencies within a population) and that it must thus manifest itself as change in the genome sequence. Which genes and thus trait functions are then associated with fast adaptations and are thus likely the target of selection? Which specific molecular mechanisms generate the necessary changes within the genome (Stapley et al. 2010)? Is adaptation possible through changes in a variety of different genes or are such changes limited to only one or few genes, resulting in convergent evolution (Dettman et al. 2012)? These questions can now be efficiently addressed with the help of whole-genome sequencing of evolved experimental populations (Hegreness and Kishony 2007; Toprak et al. 2011).

Here, we expand the data from our previous study on the experimental evolution of *E. coli* antibiotic resistance (Peña-Miller et al. 2013) by including an additional high-dosage combination evolution treatment and newly generated genome data. On the basis of genome sequences for a total of 63 evolved populations, our aim was to address the following three questions: 1) Which trait functions, genes, and/or molecular mechanisms show patterns of convergent evolution in the resistant populations and are thus potentially adaptive (cf. Christin et al. 2010; Wake et al. 2011)? 2) Are there differences in the response to different antibiotic selection intensities (e.g., low versus high concentrations of the antibiotic combination used)? 3) What is the importance and stability of the previously observed sequence amplification (Peña-Miller et al. 2013) during resistance evolution, especially for the newly considered high-dosage combination treatment?

## Materials and Methods

### Materials

**Fig. 1.—:**
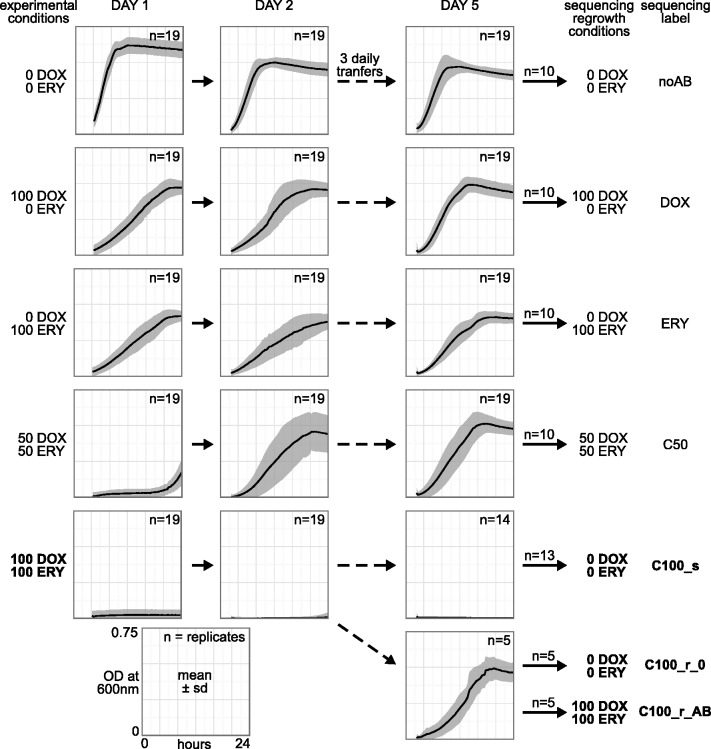
Overview of treatments, phenotypic results, and sequencing scheme. Antibiotic conditions are shown for the evolution experiment and for regrowth of replicates before whole-genome sequencing. Inset graphs are optical density (OD) measurements at 600 nm for the duration of 1 day, averaged over all replicates of a treatment and displayed with the treatment’s standard deviation as a gray band (see explanatory panel in bottom left corner). The newly considered high-dosage combination treatment C100 (with labels set in bold) diverges into two phenotypes by day 5: Five replicates become resistant and grow, whereas the other 14 replicates show no growth. The 13 viable nonresistant replicates were regrown for sequencing without antibiotics in the medium (label C100_s), the five resistant replicates were each regrown once without (label C100_r_0) and once with the original antibiotic concentrations (label C100_r_AB). Ten samples each of all the other treatments were regrown under the respective experimental antibiotic concentrations. 100 DOX = 0.15 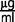; 100 ERY = 9 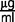. For each drug alone, these concentrations initially inhibited growth at 50% compared with the no drug control, whereas growth inhibition of the C50 combination treatment was initially close to 100% (Peña-Miller et al. 2013) due to synergy of the drugs. The high-dosage combination treatment C100 initially achieved full growth inhibition.

We used whole-genome sequencing data for independent replicate populations from our previously published evolution experiment (Peña-Miller et al. 2013). Genome data were available for four different antibiotic treatments and a control treatment without antibiotics (noAB). The two single drug treatments (doxycycline [DOX] and erythromycin [ERY]) were each calibrated to 50% growth inhibition compared with the noAB control, and the low-dosage combination treatment (C50) contained 50% of each of the single drug dosages (fig. 1). Now, we additionally considered the high-dosage combination treatment containing 100% of the single drug dosages (C100), which fully inhibited bacterial growth on day 1 (fig. 1). An initial analysis of the sequence data for all but the C100 treatments was already presented in Peña-Miller et al. (2013) but was strictly focused on the context of the respective mathematical models and their interpretation. Our new analyses used the same raw data and combined it with the sequencing data from the C100 populations and the ancestral strain of *E. coli*. The sequencing data were generated in identical form for all populations (Peña-Miller et al. 2013). Prior to sequencing, cultures were regrown for 1 day under exactly the same treatment conditions as those used during the evolution experiment. The only exception referred to the C100 replicate populations. Here, the five resistant C100 populations were each regrown twice, once with the experimental antibiotic concentrations (C100_r_AB) and once without antibiotics (C100_r_0). Of the remaining 14 susceptible populations, only 13 were viable and thus regrown to sufficient quantities in the absence of antibiotics (C100_s). Precise details on the evolution experiment, culturing conditions, DNA isolation, and next generation sequencing are provided in the supplementary material, Supplementary Material online (see also Peña-Miller et al. 2013).

### Reference Tailoring

Initially, the closest published *E. coli* K12 reference genome (BW2952 with GenBank accession NC_012759.1 in its version from the November 14, 2011, Ferenci et al. 2009) was used for read mapping and variant calling. The current reference version is available under National Center for Biotechnology Information (NCBI) accession number NC_012759. Using gap5 (Bonfield and Whitwham 2010) from the Staden package, we conducted a detailed visual review of variant calls based on the BW2952 reference (for the version number used, see supplementary table S1, Supplementary Material online). This analysis revealed the incorrect placement of sequence reads for the evolved populations, especially in regions containing breakpoints for SVs, which represent differences between the BW2952 reference and our starting strain (for a strain ancestry review, see supplementary fig. S1, Supplementary Material online). In turn, read misplacement produced erroneous single-nucleotide variant (SNV) calls with SNVer (Wei et al. 2011) and VarScan (Koboldt et al. 2012) and also erroneous breakpoint calls by Pindel (Ye et al. 2009). To minimize the number of such false calls, a tailored reference (MYMC4100) was created from the BW2952 reference and used for all read mapping, variant calling, and variant annotation (see further details in the supplementary methods, Supplementary Material online).

### Genome Analyses

With the help of the tailored reference genome, we analyzed the distribution of different types of sequence changes (i.e., SNVs, indels, SVs, and the large-scale amplification) across evolution treatments. The initial steps of the analysis followed the previously described protocol (Peña-Miller et al. 2013) as explained in the supplementary material, Supplementary Material online. For the 316-kb sequence amplification region, the average relative coverage was computed for each sequenced population using the following procedure: We first computed the absolute coverage of each sequence position and then separately averaged over all positions from the amplified region and over those from the rest of the genome. The ratio of these two gives the relative coverage for the sequence amplification region, with values above 1 indicating amplification. Because entire populations were sequenced, these coverage values reflect the average amplification level for each population. The treatment means of these population averages were then compared (fig. 3 and supplementary table S3, Supplementary Material online). For SVs and indels, Pindel (Ye et al. 2009) calls with more than ten reads support were further analyzed, thus acknowledging the conservative detection approach of Pindel (for an overview of all bioinformatics tools used, see supplementary table S1, Supplementary Material online). SNVs were only considered for further analyses if they either occurred in two independent replicate populations (irrespective of whether they were from the same or different evolution treatments) or if they were identified by both SNV callers (SNVer by Wei et al. [2011] and VarScan by Koboldt et al. [2012]). Variants also found in the no drug control were excluded from further analyses, because they very likely represent adaptations to the general experimental conditions and not to specific antibiotic treatments.

The possible function of the thus identified genes and genomic regions were inferred using the available literature and the EcoCyc database (Keseler et al. 2011; functions summarized in table 3). We additionally used the program PolyPhen2 (Adzhubei et al. 2010) for an assessment of the effect of nonsynonymous SNVs, and the online tool insertion sequence (IS) Finder (Siguier et al. 2006) for identification, confirmation and analysis of IS events, IS names, and ISs. Further details and discussion regarding known gene functions are provided in the supplementary information, Supplementary Material online.

For the documentation of synonymous SNVs as well as indels and SVs, we identified the need of a unified nomenclature of sequence changes, which is not based on amino acid changes (as is commonly used for nonsynonymous SNVs). We therefore made use of the existing Human Genome Variation Society nomenclature for sequence variants (den Dunnen and Antonarakis 2000 and see: http://www.hgvs.org/mutnomen/, last accessed May 28, 2014; used in supplementary table S4, Supplementary Material online).

## Availability of Supporting Data

Supplementary material, Supplementary Material online, is available online alongside this article and provides additional data and information—especially a detailed description of the material and methods used for genome data analysis. It additionally contains one figure and five tables: supplementary figure S1, Supplementary Material online, gives an overview of the ancestry of the *E. coli* K12 strain MC4100. Supplementary table S1, Supplementary Material online, lists all programs used for genome data analysis, including the respective version numbers. Supplementary table S2, Supplementary Material online, summarizes how the refined pipeline and the tailored reference genome improved SNV calling. Supplementary table S3, Supplementary Material online, shows the statistical results on the variation in sequence amplification across treatment groups. Supplementary table S4, Supplementary Material online, describes the variants identified across treatments. Supplementary table S5, Supplementary Material online, summarizes the four synonymous SNVs.

We submitted our tailored *E. coli* K12 strain MC4100 reference under the name MYMC4100 to the European Nucleotide Archive (ENA) under accession HG738867 (study accession is PRJEB4621). We also deposited the original genomic DNA Illumina sequence data to the ENA sequence read archive under accession number PRJEB4687, with submitted read files named according to treatment abbreviations used throughout this article (see e.g., fig. 1).

## Results

### Phenotypic Resistance Evolution

We previously demonstrated that antibiotic resistance evolved rapidly within 2 days of the evolution experiment in both monotherapies and the C50 combination treatment (resistance sensu lato, defined as increased growth rate in the presence of antibiotic(s) relative to the ancestral control; fig. 1 [Peña-Miller et al. 2013]). Of these treatment groups, the C50 combination led to a lower bacterial growth than the single drug treatments (DOX and ERY) on day 1 only, whereas growth increased from day 2 onward, suggesting more rapid evolution of resistance in the C50 combination treatment (Peña-Miller et al. 2013). Now, we asked how bacteria respond to an even higher, above minimal inhibitory concentration dosage in the combination treatment. For this, we included a treatment where bacteria had evolved at twice the C50 concentrations of the drugs (C100 high-dosage combination treatment in fig. 1). This treatment resulted in full growth inhibition in 14 out of 19 replicate populations across the 5-day evolution period (C100_s in fig. 1). In contrast to the latter populations and the ancestral control, the remaining C100 populations were able to resume growth, strongly indicating resistance evolution (C100_r_0 and C100_r_AB, day 1 vs. day 5 in fig. 1). The additional consideration of this treatment allowed us to contrast genomic changes in 1) resistant populations subjected to drug combinations with a substantial difference in selection intensity (C50 vs. C100_r); 2) C100 populations that either evolved or did not evolve resistance (C100_r vs. C100_0); and 3) resistant C100 populations regrown with or without antibiotics prior to sequencing (equivalent to sustained versus relaxed selection for resistance; C100_r_AB vs. C100_r_0).

### Variant Calling Using a Tailored MYMC4100 Reference Genome

Our starting strain (*E. coli* K12 strain MC4100) differed from the closest published reference (BW2952, GenBank accession NC_012759.1 Ferenci et al. 2009) in five structural variants (SVs), seven insertions/deletions shorter than 50 bp (indels), and 13 SNVs. These differences likely arose during independent laboratory maintenance of the two strains (see information on strain histories in supplementary fig. S1, Supplementary Material online). To improve variant calling, we here tailored the available BW2952 reference to our starting strain, compared different variant calling tools, refined our confidence criteria, and manually inspected unannotated variants (see Materials and Methods and the supplementary material, Supplementary Material online). These alterations led to identification of 14 SNVs that we failed to detect during our previous analysis, whereas four of the previously identified SNVs could not be validated (supplementary table S2, Supplementary Material online). As a consequence, a total of 21 changes were now recorded during adaptation to only DOX, 25 changes to only ERY, 14 to the C50 combination treatment, 14 for the nonresistant C100 samples (C100_s), two for the resistant C100 samples regrown without antibiotics for sequencing (C100_r_0), and three for the resistant C100 samples regrown with antibiotics for sequencing (C100_r_AB; tables 1 and 2, and supplementary table S4, Supplementary Material online).

**Table 1:** Overview of the Number of Different Variant Types

Variant Type	Non-CDS	CDS	Total
Unique SNVs	1	29 (25 non-SYN)	30
Occurrences	1	108 (73 non-SYN)	109

	Non-CDS	CDS	Total
Unique indels	1	10 (9 frames)	11
Occurrences	6	14 (13 frames)	20

	Non-CDS (IS)	CDS (IS/DUP^a^/DEL/INV)	Total
Unique SVs	1 (1)	5 (2/1/1/1)	6^a^
Occurrences	1^b^ (1)	9 (6/1/1/1)	10^a^^,^^b^

Totals	Non-CDS	CDS	Total
Unique variants	3	44^a^	47^a^
Occurrences	8^b^	131^a^	139^a^^,^^b^

Note.—DEL, deletion; DUP, duplication; frame, frameshift; INV, inversion; SYN, synonymous.

^a^Value excludes the large-scale sequence amplification.

^b^Value considers only one occurrence of the *lon* variant, as it occurred in two nonindependent samples from the same replicate population that were regrown differently before sequencing.

**Table 2:** Distribution of Independent Mutational Changes (SNVs/Indels/SVs) across Evolution Treatments and Affected Genes

Gene	DOX^a^	ERY^a^	C50^a^	C100_s^a^	C100_r_0^a^	C100_r_AB^a^	Total No. of Pops^b^
*acrA*				1/0/0			1
*acrB*		1/0/0					1
*acrR*		2/0/0		2/0/0			4
*clcB*		1/0/0					1
*dnaQ*	1/0/0	1/0/0	2/0/0				4
*frmR*		0/1/0	0/1/0	0/1/0		0/3/0	6
*ftsP*	2/0/0	2/0/0					4
*lon*					0/0/1	0/0/1	2^c^
*marR*	4/4/1			5/3/1			16
*mdaB*	1/0/0						1
*melR*	4/0/0	5/0/0	7/0/0				16
*menC*			0/0/1	0/0/1			2
*mngB*	2/0/0		2/0/0				4
*nudC*		2/0/0	3/0/0				5
*qor*	5/0/0	3/0/0	5/0/0				13
*rcnA*			1/0/0				1
*recO*	6/0/0	2/0/0	5/0/0				13
*ycbZ*		3/4/4		0/1/0			8
*ydhW*	2/0/0						2
*yjjG*	3/0/0	4/0/0	5/0/0				12
*yjjU*	1/0/0	1/0/0	4/0/0				6
*yohF*	1/0/0	2/0/0	2/0/0				5
*ypfI*		2/0/0	1/0/0				3
amplif^d^	0/0/3	0/0/3	0/0/9	0/0/8	0/0/5	0/0/5	33
Total no.^e^	10/4/4	10/5/5	10/1/9	6/5/9	0/0/5	0/3/5	36/18/37
Combined total^f^	10	10	10	12	5	5	52
Total *N*^g^	10	10	10	13	5	5	53

^a^The three digits represent the number of populations with SNVs/indels (<50 nt)/SVs.

^b^Total no. of pops, number of replicate populations affected by variants in the respective gene.

^c^These two samples were derived from the same replicate population with different regrowth conditions for sequencing (fig. 1).

^d^amplif, 316-kb amplification containing *acrAB*.

^e^Total no., number of affected replicate populations per treatment and variant type.

^f^Combined total, number of replicate populations per treatment with any variant.

^g^Total *N*, total number of sequenced populations per treatment.

### Sequence Amplification

**Fig. 2.—:**
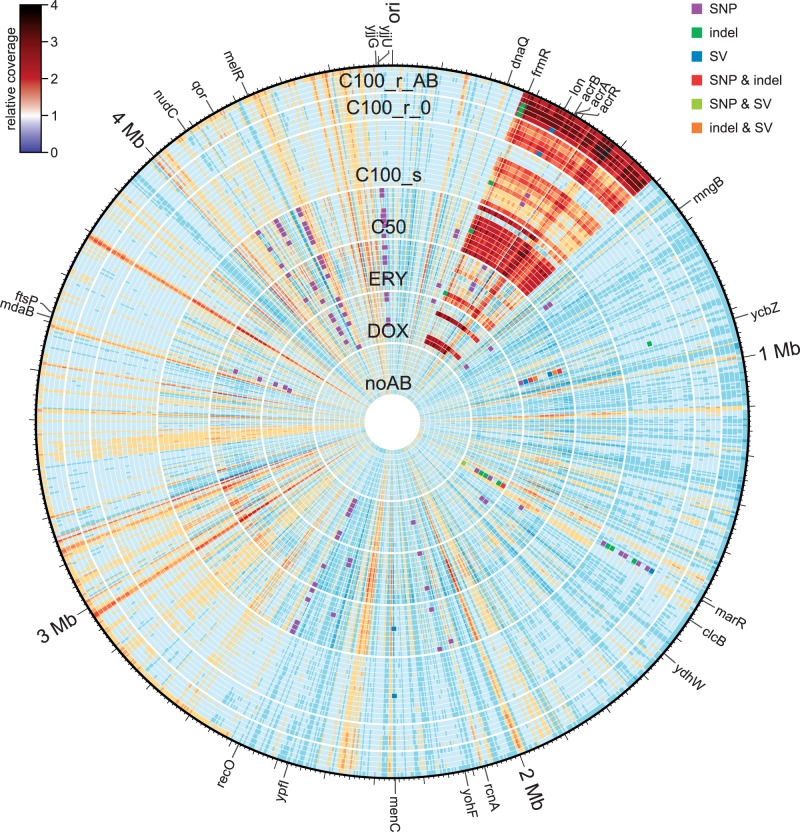
Sequence coverage and location of sequence variations across replicate populations and treatments. Each circular coverage plot represents one population of the respective treatment. Colored squares indicate different types of mutational changes in the various replicate populations, with the affected genes marked on the outer ring (i.e., outside the genome position scale). Combination treatments show a higher prevalence of sequence amplification, especially under high dosage conditions, and contain a smaller number of other variants.

**Fig. 3.—:**
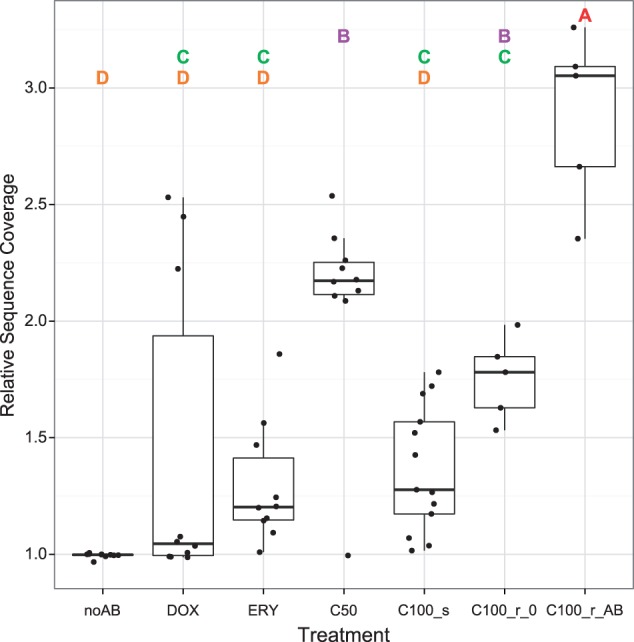
Average sequence coverage for the amplified region across treatments. Each dot represents the relative coverage of the whole 316-kb fragment per replicate population. Relative coverage is always normalized to the average genome coverage of the respective replicate (calculated excluding the 316-kb region). Treatment labels are as specified in figure 1. Treatments not sharing a common letter (placed at the top) significantly differ in their average coverage based on a Tukey HSD test (supplementary table S3, Supplementary Material online).

We could confirm our previous finding that the duplication of a large genomic region is significantly associated with rapid resistance evolution in the C50 combination treatment (figs. 2 and 3; Peña-Miller et al. 2013). This amplification of a 316-kb sequence region contains numerous known resistance genes such as those coding for components of the AcrA-AcrB-TolC efflux pump. Our previous repetition of the evolution experiment with an *acrAB* knockout strain indeed suggested that duplication of the *acrAB* operon directly contributes to fast adaptation (Peña-Miller et al. 2013). The same sequence amplification was now found in the newly sequenced populations from the C100 treatment (figs. 2 and 3). The degree of sequence amplification (i.e., the copy number of the respective chromosomal segment) varied depending on the treatment and also growth conditions prior to sequencing. Although none of the antibiotic-free controls (noAB) and only few samples of the single drug treatments (DOX and ERY in figs. 2 and 3) showed clear signs of sequence amplification, a significant increase was found for both the C50 and the resistant C100 samples (C100_r_0 and C100_r_AB; figs. 2 and 3; supplementary table S3, Supplementary Material online). Moreover, the resistant C100 samples that were regrown in the presence of antibiotics prior to sequencing (C100_r_AB) had a significantly higher average level of sequence amplification (around 3-fold) than all other groups—notably including both the C50 and the C100_r_0 groups. In contrast, the average amplification level for the susceptible C100 samples (C100_s) was only slightly elevated and thus significantly lower than those of the C100_r_AB and C50 treatments (figs. 2 and 3; supplementary table S3, Supplementary Material online). Interestingly, the start and end points of the amplified region were always located within the same two copies of the IS gene *insH* (compare e.g., Nicoloff et al. 2007; Adler et al. 2014).

### SNVs, Indels, and SVs

We identified 47 changes across the evolved populations (in addition to the above reported sequence amplification). Forty-four of these affected coding sequences (CDSs), whereas the remaining three changes (one SNV; one insertion/deletion, indel; and one IS event) fell into known regulatory sequences (table 1 and supplementary table S4, Supplementary Material online). A similar pattern emerged for the frequency of variant occurrences (i.e., the total number of times, sequence changes were found across all replicate populations), for which 131 out of 139 cases fell into CDSs (table 1). In total, 23 genes were affected by mutational changes (for their functions see table 3), which encompassed all three main types of sequence alterations (SNVs; indels; and SVs). In particular, 30 unique SNVs occurred a total of 109 times across all but two treatments (tables 1 and 2, and supplementary table S4, Supplementary Material online). The two treatments without SNVs, the resistant C100 populations regrown either with or without antibiotics (C100_r_AB and C100_r_0), only showed SVs including the large-scale sequence amplification. Twenty-nine out of the 30 unique SNVs were found in CDSs (a total of 108 occurrences), and all but four resulted in nonsynonymous changes (table 1 and supplementary table S4, Supplementary Material online). Interestingly, the four synonymous SNVs were present 35 times, representing approximately a third of all SNV occurrences (table 1 and supplementary tables S4 and S5, Supplementary Material online).

**Table 3:** Known Functions of Genes Affected by Variants

Genes and Functions
*acrA* encodes the component of the AcrA-AcrB-TolC efflux pump (Blair and Piddock 2009; Symmons et al. 2009) which spans the perisplasmic space connecting AcrB and TolC (Ma et al. 1993, 1995; Zgurskaya and Nikaido 1999, 2000; Higgins et al. 2004; Mikolosko et al. 2006; Symmons et al. 2009).
*acrB* encodes the inner membrane pump (Eicher et al. 2009) part of the AcrA-AcrB-TolC efflux pump (Blair and Piddock 2009). It is responsible for substrate specificity (Elkins and Nikaido 2002).
*acrR* encodes the repressor of the *acrAB* operon (Ma et al. 1996). It can bind a variety of structures in its multi-entrance binding pocket (Li et al. 2007; Su et al. 2007; Routh et al. 2009) and disruption of AcrR increases AcrA (Wang et al. 2001) and AcrB levels (Webber and Piddock 2001).
*clcB* encodes a putative voltage-gated chloride channel, inferred by homology to *clcA* (Accardi and Miller 2004). ClcA in turn is implied to be involved in acid resistance (Iyer et al. 2002).
*dnaQ* encodes the 3’-5’ exonuclease of DNA polymerase III, responsible for fidelity in DNA replication (Scheuermann et al. 1983). Disruption leads to a transversion mutator phenotype (DiFrancesco et al. 1984; Wu et al. 1990).
*frmR* encodes a transcriptional repressor of the *frmRAB* operon (Herring and Blattner 2004), whose products FrmA and FrmB serve to detoxify formaldehyde (Gutheil et al. 1992; Gonzalez et al. 2006). FrmR is part of the CsoR-like_DUF156 superfamily (Liu et al. 2007) of transcriptional regulators, some of which are involved in multidrug sensing (Liu et al. 2007).
*ftsP* (Kato et al. 1988) encodes for a stabilizer of divisome assembly under stress conditions (Samaluru et al. 2007).
*lon* encodes the Lon protease, responsible for MarA (and SoxS) turnover (Nicoloff et al. 2006; Nicoloff and Andersson 2013) and the variant found here has been shown to increase IS activity (Nicoloff et al. 2007) and facilitate duplications involving *acrAB* (Nicoloff and Andersson 2013).
*marR* encodes the repressor (Seoane and Levy 1995; Maneewannakul and Levy 1996) of *marA*, which in turn regulates *acrAB* expression (Barbosa and Levy 2000).
*mdaB* encodes an NADPH-specific quinone reductase (Hayashi et al. 1996), involved in a quinone redox cycle in *E. coli* (Adams and Jia 2005).
*melR* encodes a regulator of the *melAB* operon (Hanatani et al. 1984), with MelA an alpha-galactosidase (Schmitt 1968; Burstein and Kepes 1971; Nagao et al. 1988) and MelB a cotransporter of a cation (H*^+^*, Na*^+^*, Li*^+^*) and certain sugars (among them melibiose) (Yazyu et al. 1984; Wilson DM and Wilson TH 1987; Reizer et al. 1994; Wilson and Ding 2001).
*menC* (Sharma et al. 1993) encodes the O-succinylbenzoate synthase (OSBS) (Palmer et al. 1999; Thompson et al. 2000). This enzyme is part of the menoquinone biosynthesis pathway (Bentley and Meganathan 1982). The resulting menoquinone (or vitamin K2) is necessary for anaerobic growth (Newton et al. 1971).
*mngB* (recently renamed from *ybgG*) encodes an alpha-mannosidase (Sampaio et al. 2004)
*nudC* encodes a member of the nudix hydrolase superfamily (McLennan 2006), thought to be involved in sustaining oxidation under anaerobic conditions (Frick and Bessman 1995; Bessman et al. 1996).
*qor* encodes an NADPH:quinone oxidoreductase possibly involved in quinone detoxification (Lilley et al. 1993; Edwards et al. 1994; Thorn et al. 1995; Bolton et al. 2000; Ross 2004)
*rcnA* encodes a nickel and cobalt efflux protein (Rodrigue et al. 2005). Its repressor RcnR is in the same superfamily as FrmR (see above).
*recO* encodes a protein that is part of the homologous recombination RecF pathway (Kolodner et al. 1985; Morrison et al. 1989), which is responsible for the repair of stalled or broken replication forks by homologous recombination (Cox 2007).
*ycbZ* encodes a putative peptidase with domains homologous to a Lon protease domain (see http://www.uniprot.org/uniprot/C4ZQ81, last accessed May 28, 2014), suggesting similar functionality.
*ydhW* is predicted to encode part of an oxidoreductase, probably activated under anaerobic growth conditions (Partridge et al. 2008).
*yjjG* encodes a nucleotidase from the haloacid dehalogenase (HAD)-like superfamily showing phosphatase activity on dTMP, dUMP, and UMP (Proudfoot et al. 2004; Kuznetsova et al. 2006) and protects DNA against the potentially mutagenic incorporation of noncanonical pyrimidine derivatives (Titz et al. 2007).
*yjjU* encodes a putative transcriptional regulator (Serres et al. 2001) and is inferred by sequence homology to be a lipid hydrolase (see http://www.uniprot.org/uniprot/P39407, last accessed May 28, 2014). It is upregulated when mitomycin C causes DNA damage in cells (Khil and Camerini-Otero 2002).
*yohF* encodes a putative acetoin dehydrogenase (diacetyl reductase) (Reed et al. 2003).
*ypfI* (recently renamed to *tmcA*), encodes an enzyme which specifically acetylates the wobble base of *E. coli* elongator tRNA(Met) (Ikeuchi et al. 2008) which is required for correct AUG codon recognition.

In addition to SNVs, 11 unique indels (non-SNVs shorter than 50 nucleotides) were identified (tables 1 and 2; supplementary table S4, Supplementary Material online). Only one single-nucleotide indel fell outside of CDSs, affecting the regulatory sequence of *frmR*. Nine additional single-nucleotide indels were found in CDS, where they caused a frameshift and were thus nonsilent. The remaining indel produced a deletion of four complete codons in *marR* in only one of the DOX single drug treatments (DOX; supplementary table S4, Supplementary Material online). We further detected six unique SVs (in addition to the large-scale sequence amplification), consisting of three unique IS events, one duplication event, one deletion event, and one inversion event (tables 1 and 2, supplementary table S4, Supplementary Material online).

The distribution of sequence changes showed distinct differences among the evolution treatments (table 2 and supplementary table S4, Supplementary Material online; fig. 2). In the single drug treatments (DOX and ERY), all populations contained SNVs and approximately half of them indels and/or SVs. The C50 treatment also had SNVs in all populations. However, it differed from the single drug treatments regarding the other variant types, as only one of the C50 populations was affected by indels, but nine out of ten by the large sequence amplification. This pattern was even more pronounced in the high-dosage combination treatment C100: Of the 13 sequenced susceptible C100_s populations, only six contained SNVs, five indels, but nine were affected by SVs. The resistant C100 populations lacked SNVs altogether, although three out of five samples regrown with antibiotics for sequencing (C100_r_AB) had indels and all five samples from both regrowth conditions (C100_r_0 and C100_r_AB) showed SVs including the large amplification.

### Convergent Evolution

The independently evolved replicate populations showed sequence variations with identical or related functional consequences, strongly suggesting convergent evolution. In particular, presence of the large sequence amplification was significantly enriched in the C50, C100_r_0, and C100_r_AB treatments (figs. 2 and 3; table 2, and supplementary tables S3 and S4, Supplementary Material online). For the DOX monotherapy and the nonresistant C100_s treatment, independent sequence changes specifically accumulated in *marR* (a total of 9 SNVs, 7 indels, and 2 SVs; fig. 2; table 2 and supplementary table S4, Supplementary Material online). Changes in the ERY populations similarly fell in only few genes, especially *ycbZ* (a total of three SNVs, four indels, and four SVs; fig. 2; table 2 and supplementary table S4, Supplementary Material online).

**Fig. 4.—:**
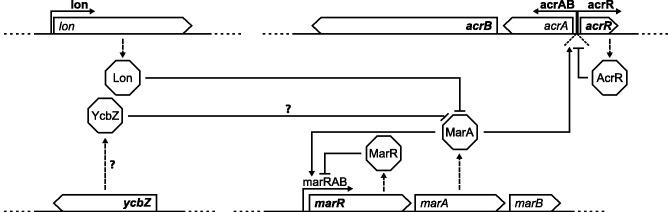
Components of the AcrA-AcrB-TolC efflux pump regulon. Bold labels point to genes or regulatory sequences with mutational changes in the evolved populations that are either known or likely to affect gene function, as indicated. Gene lengths are drawn to scale, with *mar*-genes depicted at ×4 magnification compared with all others.

Interestingly, convergent changes were observed across different organizational levels, affecting either the same SNV at a particular nucleotide position, the same synonymous mutation within a particular codon, the same gene, or the same functional unit. For instance, exactly the same SNV in the gene *melR* was found in 16 independent replicate populations of the DOX, ERY, and C50 treatments (table 2 and supplementary table S4, Supplementary Material online). Most impressively, a particular synonymous SNV was identified to cause the same Glycine codon change (GGC to GGG) in four unrelated genes (*mngB*, *qor*, *recO*, and *yohF*) for a total of 35 cases across the DOX, ERY, and C50 treatments (fig. 2, table 2, supplementary tables S4 and S5, Supplementary Material online). Several genes also showed an accumulation of different types of mutational changes, especially the genes *marR* and *ycbZ* (see above, fig. 2 and table 2). At an even broader level, two main types of functions were particularly affected by sequence changes across the involved genes: 1) the AcrA-AcrB-TolC efflux system (e.g., genes *acrA*, *acrB*, *acrR*, *lon*, *marR*, and *ycbZ*; see fig. 4), supporting its prominent role in mediating antibiotic resistance, even in populations without the large-scale sequence amplification and 2) DNA integrity (e.g., *dnaQ*, *lon*, *recO*, and *ycbZ*). For these two types of functions, convergence is particularly common across the independent populations from the DOX, ERY, and C50 treatments (fig. 2, table 2, and supplementary table S4, Supplementary Material online).

## Discussion

In this study we analyzed the genomes of 63 available *E. coli* populations that rapidly evolved resistance to different antibiotic treatments under tightly controlled experimental conditions (Peña-Miller et al. 2013). A specifically tailored reference genome was used for reliable variant identification, revealing several distinct genomic sequence changes which associate with fast bacterial adaptation. Most crucially, our analyses included high numbers of independent replicate populations, allowing us to elucidate convergent patterns of rapid molecular evolution.

### Resistance by Amplification of a Large Sequence Region Including Multidrug Efflux Pump Genes

The highly resistant populations in our experiment harbored an amplification of a 316-kb region, thus confirming our own previous results (Peña-Miller et al. 2013) and the identified genetic basis of evolved *E. coli* tetracycline resistance in a previous selection experiment (Nicoloff et al. 2006, 2007). The amplified region contains the *acrAB* operon (Peña-Miller et al. 2013), which encodes two components of the AcrA-AcrB-TolC multidrug efflux pump (Ma et al. 1995; Nishino and Yamaguchi 2001; Sulavik et al. 2001; Blair and Piddock 2009; Symmons et al. 2009). The importance of such membrane pumps for multidrug resistance has not only been shown for bacteria (Nikaido and Takatsuka 2009) but also for fungi (Cannon et al. 2009), malaria (Sanchez et al. 2010), and human cancer cells (Gillet et al. 2007). Sequence amplification of genes encoding such pumps can increase the number of efflux pumps in the cell membrane and thus the ability of microbes to cope with antifungal drugs (Selmecki et al. 2008) or antibiotics (Sandegren and Andersson 2009). Interestingly, the genomic mechanism of sequence amplifications does not only contribute to antibiotic resistance evolution but also seems to represent a more general adaptive strategy of bacteria against highly stressful environments, as previously illustrated during carbon starvation, heat, or heavy metal stress (Andersson and Hughes 2009).

### Large-Scale Sequence Amplification Scales to Selection Pressure

As an important extension to our previous findings (Peña-Miller et al. 2013), our new analyses demonstrate that the amplification level (i.e., the copy number of the amplified chromosomal segment) is significantly higher when drug dosage and thus selection intensity is increased (in the C100 treatment compared with C50). Interestingly, resistance evolution over the 5 days of our experiment is less frequent under the higher dosage combination treatment (5 out of 18 sequenced C100 populations compared with 10 out of 10 in C50) and appears asynchronously in C100, when compared with the C50 populations developing resistance almost in unison. This suggests that a higher drug dosage selects for a copy number above two, which is much less probable and requires a longer waiting time. This is very likely connected to 1) the molecular mechanism of sequence amplification and 2) differential initial growth capabilities in the different combination treatments. The location of the amplification breakpoints in two identical IS gene copies (*insH*) suggests homologous recombination to be the main duplication mechanism (e.g., Roth et al. 1996; Lovett 2004; Hastings et al. 2009). Assuming such a mechanism, a duplication only requires one step of amplification, whereas a triplication would require two consecutive steps. As 3–10% of cells in any population are estimated to bear a duplication of some chromosomal segment (Anderson and Roth 1981; Roth et al. 1996), the *acrAB* containing duplication is probably present in the inoculation culture at high enough frequencies to be transferred into all replicate populations of the experiment. This enables populations in C50 replicates to almost immediately resume exponential growth. In the C100 treatment, cells with the same duplication would have an increased chance of survival but would require a further recombination event to resume normal growth—which is probably the reason for the asynchronous waiting times. In addition, most recombination mechanisms rely on replication of the chromosome, which is only initiated once per cell cycle (see e.g., Mott and Berger 2007) and further amplifications should therefore only appear in growing populations. As C100 populations do survive, but initially show no visible growth, the necessary amplification event is even less likely, providing a further explanation for the lower abundance of resistant populations and the different waiting times.

### Large-Scale Sequence Amplification Is Unstable

When the highly resistant C100 populations with 3-fold amplifications were regrown in the absence of antibiotics (C100_r_0), amplification levels dropped significantly within 24 h (fig. 3). A similar effect was recently found, where an adaptive sequence amplification in *E. coli* was shown to entail a fitness cost (Adler et al. 2014). This suggests that amplifications generally come at high fitness costs and are lost from the population when favorable selection subsides (e.g., because drug treatment ceases) and/or when other types of resistance mutations (e.g., based on SNVs) occur and spread through the bacterial populations (Andersson and Hughes 2009; Sandegren and Andersson 2009; Adler et al. 2014).

### Convergent Functional Targets in Resistance Evolution: AcrA-AcrB-TolC Efflux Pump and Genome Integrity

The general importance of the 316-kb amplification for fast adaptation is especially emphasized by the convergent patterns found across the independent replicate populations. Almost all resistant multidrug (C50 and C100) and some monotherapy populations (DOX and ERY) contained the sequence amplification. Such patterns of convergent evolution (convergent evolution as defined by Arendt and Reznick [2008]) are usually taken as a strong indication for the particular adaptive value of the identified mechanism and/or affected function (Christin et al. 2010; Wake et al. 2011)—irrespective of convergence being due to selection on standing or de novo sequence variation. In our case, convergent sequence amplification was mainly found in the multidrug treatments, especially when selection intensity is high. Interestingly, our new refined analyses demonstrate that convergent evolution of the *acrAB* locus is not restricted to the amplification and also not to the multidrug treatments. In populations lacking the amplification, mutational changes were identified in the same locus and in genes from the same regulon (figs. 2 and 4; table 2 and supplementary table S4, Supplementary Material online). Two of the affected genes, *acrA* and *acrB*, encode components of the AcrA-AcrB-TolC efflux pump (Ma et al. 1993, 1995; Elkins and Nikaido 2002; Eicher et al. 2009; Symmons et al. 2009). Four additional genes likely influence regulation of *acrAB* (*acrR*, *marR*, *lon,* and possibly *ycbZ*; fig. 4 [Seoane and Levy 1995; Ma et al. 1996; Maneewannakul and Levy 1996; Barbosa and Levy 2000; Nicoloff et al. 2006]). Moreover, two of the four genes (*marR* and *ycbZ*) are affected by a particularly high number of mutational changes, further emphasizing their possible adaptive value.

Our new results additionally revealed convergent evolution in genes involved in the maintenance of genome integrity and repair (i.e., *lon*, *ycbZ*, *recO,* and *dnaQ*). In detail, deficiencies in the Lon protease result in the activation of ISs (Nicoloff et al. 2007), which might lead to an elevated overall mutation rate in the genome (Chao et al. 1983) and a Lon protease deficiency has explicitly been shown to facilitate sequence duplications involving the *acrAB* locus (Nicoloff and Andersson 2013). A similar function may be expressed by *ycbZ*, which shows domain homology to the Lon protease (see http://www.uniprot.org/uniprot/C4ZQ81, last accessed May 28, 2014). RecO is part of the RecF pathway involved in repair of stalled or broken replication forks (Kolodner et al. 1985; Morrison et al. 1989; Cox 2007) and might affect occurrence of large insertions, deletions, and duplications (Lovett 2004). DnaQ influences DNA polymerase III fidelity (Scheuermann et al. 1983), and its disruption leads to a transversion mutator phenotype (DiFrancesco et al. 1984; Wu et al. 1990). Mutations in these four genes may therefore prove advantageous, especially in stressful conditions (Taddei et al. 1997), where the benefits of elevated mutation rates allowing fast adaptation outweigh the costs of deleterious mutations (Sniegowski et al. 1997). In such cases, mutators may arise and will—at least transiently—constitute a significant and detectable part of the population (Tenaillon et al. 2004; Galhardo et al. 2007; MacLean et al. 2013). Such mutator phenotypes are indeed common in resistant and pathogenic clinical isolates of various bacteria (LeClerc et al. 1996; Matic et al. 1997; Oliver et al. 2000; Lindgren et al. 2003). Even though a direct increase in mutation rate in the affected samples would not be discernible in our data, the above sequence variants could still have aided adaptation to antibiotics in our experimental populations—making these genes interesting candidates for future studies on the mechanisms of resistance evolution.

### Convergence at the mRNA Level: Synonymous Glycine Codon Changes

Overall, our analyses identified patterns of convergent evolution across different levels of biological organization (see also Losos 2011). In addition to the above observations, made at the functional and gene level, our new results also show an unusual case of convergence at the mRNA level. Four synonymous SNVs (one each in *mngB*, *qor*, *recO*, and *yohF*) each occurred in 4–13 independent replicates of the DOX, ERY, and C50 treatments. Each of these SNVs changed a GGC to a GGG Glycine codon (supplementary table S5, Supplementary Material online). The distribution of this particular synonymous change across unrelated genes and independent replicate populations strongly suggests an adaptive value of the resulting codon change. A fitness effect underlying such a possible adaptive value could come from a change in mRNA stability and/or the abundance of encoded proteins, both of which are possible results of synonymous SNVs (reviewed in Plotkin and Kudla 2011; Shabalina et al. 2013). Interestingly, synonymous codon change in an antibiotic resistance gene was previously shown to associate with a fitness increase (Schenk et al. 2012). In general, however, we still lack an in-depth understanding of the role of these synonymous changes during rapid adaptation—clearly requiring further research, particularly in the context of antibiotic resistance evolution.

## Conclusions

In conclusion, our genomic analyses of 63 independently evolved replicate populations from distinct antibiotic treatment groups revealed comprehensive convergent evolution, strongly suggesting constrained evolutionary trajectories during the adaptation to antibiotics. High selection pressure during multidrug treatments, especially under the high-dosage conditions, specifically favored amplification of a large genomic region, containing known antibiotic resistance genes such as components of the AcrA-AcrB-TolC efflux pump. We identified amplification as a potent and scalable response mechanism with a high evolutionary cost, most likely leading to its transient presence in the adapting populations. Less intense selection in the single drug treatments favored convergent mutational changes in several trait functions, including the AcrA-AcrB-TolC system and DNA integrity. In addition, we discovered that synonymous SNVs are an interesting candidate for advantageous sequence changes during adaptation. Taken together, distinct selective challenges are countered by different genomic response mechanisms, each enabling continued bacterial growth in an unfavorable environment. Thus, increased antibiotic stress does not necessarily lead to bacterial elimination but rather causes a change in the set of genomic adaptations.

## Supplementary Material

Supplementary material is available at *Genome Biology and Evolution* online (http://www.gbe.oxfordjournals.org/).

Supplementary Data
