# Supplementary material for: Genomics of Rapid Adaptation to Antibiotics: Convergent Evolution and Scalable Sequence Amplification
Source: Genome Biol Evol. 2014 May 20;6(6):1287–301. doi: 10.1093/gbe/evu106 (PMC4079197; doi:10.1093/gbe/evu106)
Supplement: Supplementary Data [file supp_6_6_1287__index.html]

Genomics of Rapid Adaptation to Antibiotics: Convergent Evolution and Scalable Sequence Amplification — Supplementary Data 

# Genomics of Rapid Adaptation to Antibiotics: Convergent Evolution and Scalable Sequence Amplification

## Supplementary Data

files

**Files in this Data Supplement:**

- Supplementary Data - zip file
