# Supplementary material for: Genomics of Rapid Adaptation to Antibiotics: Convergent Evolution and Scalable Sequence Amplification
Source: Genome Biol Evol. 2014 May 20;6(6):1287–301. doi: 10.1093/gbe/evu106 (PMC4079197; doi:10.1093/gbe/evu106)
Supplement: Supplementary Data [file supp_evu106_suppl_data.zip › 2014_genomics-of-rapid-adaptation_GBE_Supplement_revised.pdf]

# Supplement – Genomics of rapid adaptation to antibiotics: Convergent evolution and scalable sequence amplification

David Laehnemann, Rafael Peña-Miller, Philip Rosenstiel,
Robert Beardmore, Gunther Jansen, Hinrich Schulenburg

## Contents

**1 Supplementary Materials** **2**

1.1 *E. coli* K12 strains MC4100 and BW2952 . . . . . . . . . . . . . . . . . . . . 2

1.1.1 Strain Histories: where MC4100 and BW2952 originate . . . . . . . . 2

1.1.2 How MYMC4100 differs from BW2952 in its DNA sequence . . . . . . 3

1.1.3 Tailoring a reference . . . . . . . . . . . . . . . . . . . . . . . . . . . 6

**2 Supplementary Genomic Methods and Analysis** **7**

2.1 Bioinformatics Tools used . . . . . . . . . . . . . . . . . . . . . . . . . . 7

2.2 Copy Number Variation Analysis . . . . . . . . . . . . . . . . . . . . . . . 8

2.3 Detailed Variant Review . . . . . . . . . . . . . . . . . . . . . . . . . . . 8

2.3.1 *acrAB* – resistance through efflux pump regulation or modification . . 8

2.3.2 Synonymous SNVs as an unexplored molecular mechanism of antibiotic resistance evolution and convergence . . . . . . . . . . . . . . . . 11

2.3.3 DNA integrity . . . . . . . . . . . . . . . . . . . . . . . . . . . . . . . 12

2.3.4 Variants by treatment specificity . . . . . . . . . . . . . . . . . . . . 13

**3 Supplementary Figures** **17**

**4 Supplementary Tables** **18**

# 1 Supplementary Materials

## 1.1 *E. coli* K12 strains MC4100 and BW2952

### 1.1.1 Strain Histories: where MC4100 and BW2952 originate

The main background of *E. coli* strain MC4100 comes from two K12 lineages, strains Hfr 3000 U169 and MO. *E. coli* K12 was originally isolated from faeces in 1922 at Palo Alto (California, USA) by Blair (Bachmann, 1996; Lederberg, 2004). The lineage has been traced by (Bachmann, 1996) as detailed as was still possible at the time and we tried to add to this reconstruction as much as we could. A summary is given in Figure 1 and all details are specified in the following:

At some point, K12 was passed on to William Hayes in England, making it Hfr Hayes (with several confusing alternative strain names, as is specified by (Bachmann, 1996): HfrH, W2323, Hfr2 Lederberg, Hfr2 or Hfr2 (Paris)). From this strain a recombinant strain was derived in Paris, called Hfr 3000 ((Bachmann, 1996) also reports alternative names used: HfrH, AB259, HfrC, and Hfr H (Paris)). The Hfr 3000 strain was then subjected to UV radiation and – among others – *E. coli* K12 strain Hfr 3000 U169 was isolated by Francois Jacob. He, in turn, probably passed it directly to Jonathan Beckwith with whom he collaborated (see Jonathan Beckwith's CV: `http://beck2.med.harvard.edu/CV%20and%20Laboratory%20publications.doc` [June 7th 2012]) and the strain was assigned the synonymous name CA7027 (according to the Coli Genetic Stock Center: `http://cgsc2.biology.yale.edu/Strain.php?ID=4947`). In parallel, another strain (MO) was derived from Hfr Hayes by Sydney Brenner, which is only documented by "personal communications" in (Hancher et al., 1969).

MC4100 was then derived from *E. coli* K12 strains MO and Hfr 3000 U169 through a series of strain constructions, which are – once again – only mentioned as "personal communications" from Beckwith in (Ferenci et al., 2009). This suggests that Beckwith was involved and maybe even conducted them himself. The steps included the transduction of the *araD-139* mutation into that background (Casadaban, 1976). This mutation stems from the *E. coli* B strain derivative B/r. The original *E. coli* strain B can now be traced back as far as 1918 to the collection of Felix D'Herelle at the Institut Pasteur (Daegelen

et al., 2009). *E. coli* B/r was then derived from *E. coli* strain B by Evelyn Witkin, who had obtained it from Delbrück and Luria. The “r” in Strain B/r refers to it being “resistant to radiation”, meaning it is less sensitive to both UV and X-ray radiation (Witkin, 1946). *E. coli* B/r was then passed on to Milislav Demerec (Demerec, 1946). Ellis Englesberg obtained the strain from Demerec and generated several *ara* mutant strains (Gross and Englesberg, 1959), of which mutant *araD-139* was later characterised to have a defect in *araD* (Englesberg et al., 1962). This mutation was then transduced into the above mentioned steps which ultimately created MC4100.

*E. coli* K12 strain BW2952, the most closely related strain with a published complete genome sequence ([GenBank:NC_012759.1]), was constructed from an MC4100 derivative kept in the Ferenci-lab. This group transduced a *malG-lacZ* fusion into MC4100 using $\lambda$p*lac*Mu50 (Notley and Ferenci, 1995) and sequenced that strain at a later time (Ferenci et al., 2009). Thus, this BW2952 strain and the MC4100 which we obtained from the Coli Genetic Stock Center (CGSC) and for which we submitted an assembled genome sequence under the strain name MYMC4100, stand in no clear line of descent. Rather, they probably have a common ancestor from which they both diverged in different ways.

### 1.1.2 How MYMC4100 differs from BW2952 in its DNA sequence

The BW2952 genome sequence (accession [GenBank:NC_012759.1], January 2012) was initially used as the reference for mapping of the reads of the starting *E. coli* K12 strain MC4100 (as we obtained it straight from the Coli Genetic Stock Center). Using several variant callers, we could identify sequence differences between our strain and the published sequence. These were as follows, where each of them is reported as if it was a sequence alteration event from BW2952 to our MC4100. But, as mentioned above, they probably rather represent events in either of the strains after divergence from the last common ancestor: Altogether, five structural variants (SVs) and seven insertions/deletions shorter than 50 bp (indels) were found by Pindel and 13 single nucleotide variants (SNVs) were reported by SNVer and VarScan (for software versions, please refer to Table 1).

Of the five SVs, two were full deletions of *insB* genes at different BW2952 reference positions (776 bp and 777 bp deleted). A further analysis with the ISFinder (Siguier et al., 2012) revealed that the respective surrounding sequences showed similarity to an *E. coli*

IS*1* insertion element (`http://www-is.biotoul.fr/index.html?is_special_name=IS1A`) – one of them with a perfect sequence match and the other with a one nucleotide mismatch. This means, that not only the *insB* gene is present in BW2952, but a whole (and probably functional) insertion sequence (IS), including the *insA* gene. This, along with information from (Ferenci et al., 2009) (Table 1), suggests that these were insertions which occured after BW2952's divergence from a common ancestor.

A third deletion removes a total of 175 nucleotides from the CDS of the gene *amiC*, deleting a considerable amount of codons and causing a frameshift for the downstream sequence of the gene. As the deletion itself removes codons coding for amino acids of an ion binding site, this deletion should render the encoded enzyme, the N-acetylmuramyl-L-alanine amidase AmiC, non-functional. The loss of its function might be compensated in parts by the N-acetylmuramyl-L-alanine amidases AmiA and AmiB (Heidrich et al., 2001), both present in MYMC4100, allowing normal growth. But nevertheless, a loss of function should be observable through a certain portion (20-30%) of the population forming chains of 3-6 cells, as they are slightly impeded in cell division (Heidrich et al., 2001). Although no quantitative measurements were taken, such chain forming behaviour was observed under the microscope. In addition, the AmiC function in autolysis under stress of a combination of beta-lactam aztreonam and bulgecin (an inhibitor of the peptidoglycan recycling enzyme Slt70) will probably be compensated by AmiA and AmiB (Heidrich et al., 2001), but the loss of AmiC function might lead to a slightly decreased sensitivity to this antibiotic combination.

The breakpoints of the only reported inversion do not annotate to any feature. However, they lie at corresponding positions of highly similar sequences of the BW2952 *rrnG* (position 2,613,172) and *rrnB* operon (position 4,056,183), two paralogous RNA genes. Around these breakpoints several SVs and SNVs were reported, which visual inspection of the alignment in gap5 (Table 1) revealed as artefacts of misaligned reads due to the inversion breakpoints.

The last deletion of 49,165 bp refers to the large insertion unique to BW2952, generated by the above mentioned transduction step in its construction ((Ferenci et al., 2009) and Figure 1).

Of the seven indels, only one single nucleotide clearly annotated to a coding gene: a

putative transporter gene (*BWG_1070*). However, further analysis with ISFinder revealed another mis-annotation of an IS: Together with the sequence of gene *BWG_1071*, which overlaps the end of *BWG_1070*, this rather is an IS*421* (`http://www-is.biotoul.fr/index.html?is_special_name=IS421`). Only four nucleotides differ from the IS*421* consensus – one of them is the here reported single nucleotide deletion. This suggests, that this deletion also rather represents the insertion of a single nucleotide in BW2952 after divergence from the last common ancestor and demonstrates, how an automated variant analysis would benefit from a thorough IS annotation in the reference genome.
Two further single nucleotide deletions and a short sequence replacement did not annotate to any functional gene. But they are located within the sequence of a Glu tRNA pseudogene *gltT*, that had already been rendered non-functional by a deletion and seems to have accumulated two more deletions in MYMC4100 or two insertions in BW2952, plus a sequence replacement in either of the strains.
The remaining small insertion (2 bp) and the two short sequence replacements reported by Pindel did not annotate to any protein coding genes, but are found in close proximity to the breakpoints of the large inversion, probably connected to the inversion event in some way, as visual inspection confirmed these variants as valid.

Two of the 13 SNVs were in paralogous genes and were both only reported by VarScan, each at 50% of the reads mapped at these positions and both of them the same synonymous SNV. This suggests the emergence of a SNV in one of the copies with the stretches of sequence similarity around the sites too long for unambiguous read pair placing.
For all other 11 SNVs the reported, alternative MYMC4100 allele is supported by 100% of the reads at the locus, meaning that the BW2952 allele is not present in the sequenced populations at all. One of the SNVs was only reported by SNVer: it is predicted to have only a "benign" effect (PolyPhen-2, Table 1) on GspD through an E160D amino acid change in the protein sequence.
The remaining 10 SNVs were all reported by both callers alike, but four of them do not annotate to any gene. Another one reports a difference in a 23S rRNA gene, where MYMC4100 probably contains the ancestral allele compared to BW2952. A further one introduces an early stop codon in *gatZ*, truncating the encoded protein. But *kbaZ* is a paralogue which can probably substitute its function (Brinkkötter et al., 2000). And one

of the further SNVs is synonymous within *yegE*, encoding a putative sensor protein. The remaining three SNVs cause non-synonymous codon changes in different genes: *deoR*, *ycjY* and *hdhA*. Of these, only the change in *deoR* is reported as a "probably damaging" SNV by PolyPhen2. This change is likely to lead to a deficiency in deoxyribonucleoside catabolism (Buxton et al., 1977; Valentin-Hansen et al., 1985; Mortensen et al., 1989; Dandanell et al., 1991; Anantharaman and Aravind, 2006), but the CGSC genotype already reports a mutation in the same operon, *deoC1*. Thus, MYMC4100 might have accumulated this SNV in a different gene of an already defunct pathway.

Also, six regions of zero coverage were found, which were not reported by Pindel as deletions. We tried to resolve these regions as follows: All reads that found no equivalent sequence in the reference (BW2952), were extracted from the bam-file using Picard (Table 1) command-line tool SamToFastq and then assembled de novo with velvet (Table 1), using the VelvetOptimizer.pl script. Most of the de novo contigs either replicated known sequences or did not overlap with any of the genome's sequence. But one of these de novo contigs spanned one of the six zero coverage regions, thus resolving its sequence with some newly inserted nucleotides (already included in the above discussion of indels). In addition, we also broke the BW2952 reference at the other five sites, resulting in five pseudo-contigs and then mapped the reads against them and visually inspected the result in gap5. This showed that there were read pairs spanning each of these zero coverage regions linking the pseudo-contigs in exactly the same order as they were in the original reference (BW2952). Thus, these remaining five zero-coverage regions are probably due to some bias in the sequencing and we were not able to resolve them. These regions plus five nucleotides to both of their sides were therefore filtered out from all further variant analysis (using BEDtools, Table 1), as coverage would have been to low for reliable calls.

### 1.1.3 Tailoring a reference

All the confirmed SVs, indels and SNVs were manually applied to the BW2952 sequence in the respective fasta file (NC_012759.fna). The genbank feature annotation file (NC_012759.gbk) was adjusted using a custom Bioperl (Stajich et al., 2002) script and the functional annotation information provided by snpEff was added by manual editing. Finally, the manually

adjusted sequence was also used to substitute the sequence in the genbank file with a Bioperl script.

Altogether, the tailoring process can be summarized as follows:

1. Mapping reads of MYMC4100 against BW2952 reference.
2. SV calling using Pindel and visual confirmation using gap5.
3. SV annotation using snpEff.
4. Integration of confirmed SVs into BW2952 reference (custom Perl script and manual editing) → intermediate reference (genbank file).
5. Mapping reads of MYMC4100 against intermediate reference.
6. SNV calling using VarScan and SNVer and visual confirmation using gap5.
7. SNV annotation using snpEff.
8. Integration of confirmed SNVs into intermediate reference (custom Perl script and manual editing) → tailored MYMC4100 reference.

The resulting local reference genome MYMC4100 comprising of a total of 4,527,247 bp is only as good as the reference it is based on and would profit from a further resolution of the zero coverage regions when using Illumina sequencing. But with fasta and genbank files for MYMC4100 accurately incorporating all variants in both the sequence and the annotations, this reference can be used for any of the further analyses. All tools accept the fasta format for sequences and most accept the genbank format for annotations. For tools that require other formats, all other major sequence and annotation formats can easily be generated from the genbank file using Bioperl or other languages' Bio-implementations.

# 2 Supplementary Genomic Methods and Analysis

## 2.1 Bioinformatics Tools used

A variety of bioinformatic tools were used for analyses of population genome sequence data, as summarized in Table 1 and outlined in more detail below.

## 2.2 Copy Number Variation Analysis

For the analysis of copy number variation, the absolute per-base read coverage was computed for each MYMC4100 genome position of each of the samples by running BEDtools (Table 1). For Figure 2, a custom Perl script computed the relative coverage by dividing each positions absolute coverage by the average of the whole genome excluding the potentially amplified genome region (MYMC4100 reference positions 274,201 - 590,257) and created bins of 3000 nt. The relative coverage was then plotted using Circos (Table 1). For Figure 3, the average of the relative coverage of the potentially amplified region (274,201 - 590,257) was computed and plotted using the R package ggplot2 (Table 1).

## 2.3 Detailed Variant Review

All variants which met the criteria for a thorough review (for the criteria, refer to the section "Genome Analyses" in the Methods of the main manuscript), are recorded in Table 4. They are sorted by the name of the respective gene they affect in either its coding or regulatory sequence.

### 2.3.1 *acrAB* – resistance through efflux pump regulation or modification

Many of the variants point to *acrAB* (compare Figure 4 of the main text), as did the above mentioned sequence amplification. While the sequence amplification likely changes the amount of AcrA and AcrB produced by providing further copies of the genes, the following variants affect efflux activity either by changing pump components or through regulating their transcription:

**Variants affecting AcrA-AcrB-TolC pump components:** In one replicate of the erythromycin only treatment, a SNV in *acrB* resulted in the amino acid substitution R717L. AcrB is one of the components of the AcrA-AcrB-TolC efflux pump (Ma et al., 1995), one of the most important multidrug resistance mechanisms in *E. coli* (Nishino and Yamaguchi, 2001; Sulavik et al., 2001). AcrB is responsible for substrate specificity (Elkins and Nikaido, 2002) and constitutes the inner membrane pump (Eicher et al., 2009). The affected replicate has the highest overall growth on day 5 of the experiment compared to all replicates

sequenced from that treatment. Also, it shows no other variants of note. This suggests a higher level of resistance (presumably caused by higher drug efflux) which is most probably caused by this very variant.

In accordance with this, R717 amino acid changes have been reported to modulate substrate specificity. However, in the two respective studies, the specific erythromycin MIC which would be of note here, was either not tested ((Middlemiss and Poole, 2004); change to Histidine) or no change in erythromycin MIC was found ((Yu et al., 2005); change to Alanine). As R717L surely affects one of two binding pockets, but not the one that erythromycin preferentially binds (Nakashima et al., 2011; Vargiu and Nikaido, 2012), this change probably has a different effect from those reported at the same position.

**Regulation of the *acrAB* transcription level:** *acrA* is affected by a regulatory SNV in one C100_s replicate. AcrA is the component of the AcrA-AcrB-TolC efflux pump which spans the perisplasmic space and interacts with both AcrB and TolC (Ma et al., 1993, 1995; Zgurskaya and Nikaido, 1999, 2000; Higgins et al., 2004; Mikolosko et al., 2006; Symmons et al., 2009). The reported SNV changes the last nucleotide of the known AcrR binding site (Su et al., 2007) upstream of the respective gene (which is also upstream of *acrR*, see below and Figure 4 in the main manuscript). This change presumably lowers the binding affinity of AcrR to this site, which in turn would lower AcrR's repression potential.

*acrR* is the gene encoding for the repressor of *acrAB* (Ma et al., 1996), able to bind structurally dissimilar substances in its multi-entrance binding pocket (Li et al., 2007; Su et al., 2007; Routh et al., 2009). Two SNVs were reported in erythromycin only replicates (causing T5A and Q78K), with the second one identical to a SNV in one C100_s replicate. Neither of these is in any of the known functional domains, but T5N amino acid changes have been reported in fluoroquinolone resistance (Wang et al., 2001; Lindgren et al., 2003). In addition, a SNV upstream of *acrR* (at a position also upstream of *acrA*, see above) in one C100_s replicate changes the last nucleotide of the AcrR binding site (Su et al., 2007). This presumably lowers the binding affinity of AcrR to this site, which in turn would lower AcrR's repression potential. As disruption of AcrR function is reported to increase AcrA (Wang et al., 2001) and AcrB levels (Webber and Piddock, 2001), this might be the expected outcome of any of the above mentioned mutations.

*marR* is the repressor (Seoane and Levy, 1995; Maneewannakul and Levy, 1996) of the *mar* operon (Alekshun and Levy, 1997). Upon de-repression, this operon produces MarA, which in turn activates *acrAB* and *tolC* and represses *ompF* (Barbosa and Levy, 2000), an outer membrane porin involved in antibiotic uptake (Nestorovich et al., 2002). In the doxycycline only treatment, two SVs, three single-nucleotide indels and eight SNVs (all non-synonymous) were found within the coding sequence of this gene. Also, in the non-resistant C100_s treatment, one SV, three single-nucleotide indels and four SNVs were reported. All SVs and indels either cause frameshifts or delete several codons at once and all the SNVs (except for one in a doxycycline replicate) are predicted as "probably damaging" by PolyPhen2. Thus, all variants seem to be disruptive and for two of the SNVs (found in two doxycycline replicates) the exact same amino acid change has even been reported to have diminished MarR activity by (Alekshun et al., 2000).

*lon* encodes a protease and Lon deficient mutants have been shown to have a lower MarA (and SoxS) turnover, leading to higher MarA levels (Nicoloff et al., 2006; Nicoloff and Andersson, 2013) and thus higher *acrAB* transcription. Here, an IS*186* element insertion into the *lon* promoter region has been found in one of the resistant C100_r_AB replicates regrown with antibiotics – the exact same insertion has been reported to cause a Lon deficiency (SaiSree et al., 2001). Such an insertion in that promoter after antibiotic selection has also been shown to increase IS activity (Nicoloff et al., 2007), including IS insertions into *acrR* and *marR* and IS*3*-/IS*5*-mediated duplications of large genomic regions containing *acrAB* (see also (Nicoloff and Andersson, 2013)).

YcbZ is predicted to be a protease through homologies to a Lon protease domain (`http://www.uniprot.org/uniprot/C4ZQ81`), which would selectively degrade unstable and abnormal proteins in an ATP-dependent manner (Vasilyeva et al., 2002) if indeed functional. Thus, rendering it dysfunctional might have a similar effect like the above Lon protease variant, although this is mere speculation. But such variants which would definitely knock out YcbZ are found in six of the erythromycin only replicates and one of the non-resistant C100_s replicates. For erythromycin, the variants consist of three single-nucleotide indels, four very likely copy-and-paste events of an *insCD* IS*3* element (Sekine et al., 1999) into the gene and a SNV introducing an early stop codon; for the non-resistant C100_s replicate it is a single-nucleotide deletion.

### 2.3.2 Synonymous SNVs as an unexplored molecular mechanism of antibiotic resistance evolution and convergence

We found four convergent synonymous SNVs (i.e. not altering the resulting amino acid sequence) affecting four genes, revealing the mRNA level as a selective target in antibiotic resistance. Each of these SNVs causes the same GGC to GGG Glycine codon change (Table 5). Even though a synonymous SNV does not alter the amino acid sequence of the encoded protein, it may nevertheless alter the cellular abundance and the functionality of the protein through translation efficiency. Here, three possible mechanisms are currently considered (Plotkin and Kudla, 2011; Shabalina et al., 2013): (i) Changes in mRNA secondary structure could affect mRNA turnover and translation speed, as unwinding of secondary structures is necessary for translation to proceed. (ii) A change between synonymous codons where the respective tRNAs occur at different frequencies in the cell could also alter translation speed. Any change in translation speed which either eliminates or introduces a ribosomal pausing may also have an effect on protein folding and post-translational modifications. (iii) Certain sequence motifs – e.g. important for translation efficiency (like the codon ramp influencing translation initiation) or mRNA stability (like RNase target sites) – can be disrupted or improved. Therefore, any of the changes in the following four genes might result in an adaptive fitness change:

(i) *mngB* (recently renamed from *ybgG*) encodes an alpha-mannosidase (Sampaio et al., 2004). Here, the synonymous codon change appears at position 348 of the coding sequence (Table 5) and in two replicates each of the DOX and ERY treatments, thus in a total of four replicates (Table 4).

(ii) *qor* encodes a 35-kDa soluble NADPH:quinone oxidoreductase which can reduce a variety of synthetic quinones (Lilley et al., 1993; Edwards et al., 1994; Thorn et al., 1995). Quinones can be cytotoxic by alkylating proteins and/or DNA or by generating reactive oxygen species, which can in turn damage various biomolecules by oxidising them (O'Brien, 1991; Bolton et al., 2000). In human cells, such quinone effects can be amended by the enzyme NQO1 (NAD(P)H:quinone oxidoreductase 1, also DT Diaphorase) (Ross, 2004), to which Qor is homologous (Thorn et al., 1995). The synonymous Glycine codon change is caused by a SNV at position 453 of the coding sequence (Table 5) in a total of 13 replicates of the single drug treatments (DOX and ERY) and the C50 treatment (Table 4).

(iii) The coding sequence of *recO* is affected by a synonymous Glycine codon change caused by a SNV at position 465 in 13 samples of the single drug treatments (DOX and ERY) and the C50 treatment. *recO* is part of the *rnc-era-recO* operon (Takiff et al., 1989) and encodes a protein that is part of the homologous recombination RecF pathway (Kolodner et al., 1985; Morrison et al., 1989). This pathway is responsible for the repair of replication forks which are stalled or broken (e.g. due to DNA damage) by homologous recombination (Cox, 2007). RecO can anneal ssDNA to complementary sequences by itself (Luisi-DeLuca and Kolodner, 1994; Kantake et al., 2002), but *in vivo* probably rather acts by the RecF pathway: Through binding of the single strand DNA (ssDNA) binding protein (SSB) – which coats ssDNA – RecO can displace SSB from the ssDNA (Inoue et al., 2008, 2011). Together with RecR it then facilitates the loading of RecA (Umezu et al., 1993; Bork et al., 2001), which is required for homologous recombination (Cox, 2007). Alterations in the abundance of RecO might then alter the frequency of the respective events of homologous recombination, which would in turn mean altered mutation frequencies.

(iv) *yohF* encodes for a putative acetoin dehydrogenase (diacetyl reductase) (Reed et al., 2003) and is under transcriptional control of RpoS (factor $\sigma^{38}$) during stationary phase growth of *E. coli* (Van Dyk et al., 1998). Here, a synonymous SNV is found in one DOX sample and in two samples each from ERY and C50 treatments.

### 2.3.3 DNA integrity

Three examples for potential mutators due to their respective role in DNA integrity have already been presented: the variant in *recO*, discussed above as a synonymous SNV and the variants in *lon* and *ycbZ*, which most likely increase IS activity, thus facilitating sequence amplifications, gene knockouts or any kind of recombination event. A fourth gene, like *ycbZ* affected in both single drug and combination treatments, is *dnaQ*. It encodes the $\epsilon$ subunit of DNA polymerase III, responsible for fidelity in DNA replication (Scheuermann et al., 1983). Mutations in this gene can result in transversion mutator phenotypes (DiFrancesco et al., 1984; Wu et al., 1990). Here, the exact same V58G change was found in one replicate each of the single drug treatments and two replicates of the C50 treatment.

### 2.3.4 Variants by treatment specificity

The high replication for each examined treatment enabled us to identify interesting patterns of treatment specificity for the variants we found. We use the following presentation of such patterns to also review any previously unmentioned genes with variants of interest.

**Variants affected in erythromycin containing replicates only:** Of the eight genes affected by variants only in treatments containing erythromycin (ERY and the combination treatments), we have already reviewed *acrA*, *acrB*, *acrR* and *ycbZ* above. A similar pattern was also found for the four genes *clcB*, *frmR*, *nudC* and *ypfI*:
(i) ClcB is a putative voltage-gated chloride channel, inferred by homology to a proven chloride proton antiporter channel gene, *clcA* (Accardi and Miller, 2004). ClcA in turn, is implied to be involved in acid resistance (Iyer et al., 2002). The SNV found in *clcB* in one ERY replicate confers an A25V amino acid change.
(ii) FrmR is a transcriptional repressor of the *frmRAB* operon, with its repression relieved through formaldehyde (Herring and Blattner, 2004). The products of the operon, FrmA (Gutheil et al., 1992) and FrmB (Gonzalez et al., 2006), serve to detoxify formaldehyde, produced e.g. during repair of alkylated DNA or RNA (Yu et al., 2006). The exact same variant, a single-nucleotide deletion 132 nt upstream of the *frmR* start codon, was found in one replicate each of the ERY, C50 and C100_s samples. It was also found in 3 of the resistant C100_r_w samples regrown with antibiotics. A connection to erythromycin is not clear. However, FrmR is in the same domain family as RcnR (`https://www.ncbi.nlm.nih.gov/Structure/cdd/cddsrv.cgi?uid=cd10153`), which in turn is part of the CsoR-like_DUF156 superfamily ((Liu et al., 2007); `https://www.ncbi.nlm.nih.gov/Structure/cdd/cddsrv.cgi?uid=cd10148`). This superfamily contains transcriptional regulators known to be involved in multidrug sensing (Liu et al., 2007).
(iii) *nudC* encodes for a member of the nudix hydrolase superfamily (McLennan, 2006). The NADH pyrophosphatase is thought to be involved in the balance of NADH/NAD+ in cells – a ratio that serves to regulate anabolic vs. catabolic activity in eukaryotes (Frick and Bessman, 1995). With NudC favouring NADH 100-fold over NAD+ as a substrate (Frick and Bessman, 1995), the depletion of NADH is thought to especially sustain oxidation under anaerobic conditions (Bessman et al., 1996). The exact same SNV found for

this gene, changing amino acid W13G, was identified in two ERY replicates as well as in three C50 replicates.

(iv) *ypfI* has recently been renamed to *tmcA*, when it was revealed to specifically acetylate the wobble base of *E. coli* elongator tRNA(Met) (Ikeuchi et al., 2008), required for correct AUG codon recognition. Any loss of function would thus result in an elevated level of translational errors, with ambiguity between tRNA(Met) and tRNA(Ile). Here, the exact same SNV (causing a V260G change) was found in two ERY replicates and in one C50 sample.

**Variants affected in doxycycline containing replicates only:** Two of the genes affected by variants only in treatments containing doxycycline (DOX and the combination treatments) have alreafy been discussed: *marR* and *mngB*. The two genes *mdaB* and *ydhW* show the same treatment pattern:

(i) MdaB is an NADPH-specific quinone reductase (Hayashi et al., 1996), involved in a quinone redox cycle in *E. coli* (Adams and Jia, 2005). This implies assistance in counteracting oxidative stress. Also, an overexpression of this gene leads to elevated resistance to a tumoricidal drug (Chatterjee and Sternberg, 1995). The SNV found in one DOX replicate causes a V39A amino acid change.

(ii) *ydhW* is predicted, by sequence homology, to be part of an oxidoreductase, probably activated under anaerobic growth conditions (Partridge et al., 2008). The respective SNV causing a V180G change was found in a single DOX replicate.

**Variants affected in single drug replicates only (DOX and ERY):** (i) FtsP (Kato et al., 1988) stabilises the divisome assembly under stress conditions (Samaluru et al., 2007). In one DOX replicate, two different alleles at the same position cause the amino acid changes D391G and D391E. The SNV causing V163G in another ERY replicate is also found in two DOX replicates.

**Variants affected in single drug replicates (DOX and ERY) and the lower concentration combination treatment (C50):** Seven genes are affected only in treatments with concen-

trations allowing for growth on day one (i.e. concentrations below the minimal inhibitory concentration – MIC). *dnaQ*, *qor*, *recO* and *yohF* have been discussed above as possible mutators and/or as synonymous SNVs. The following is known about the remaining three genes *melR*, *yjjG* and *yjjU*:

(i) *melR* encodes for a regulator of the *melAB* operon (Hanatani et al., 1984) and is situated directly upstream of and in opposite direction to the *melA* gene (Webster et al., 1987). MelA is an alpha-galactosidase (Schmitt, 1968; Burstein and Kepes, 1971; Nagao et al., 1988), MelB is a cotransporter of a cation ($H^+$, $Na^+$, $Li^+$) and certain sugars (among them melibiose) (Yazyu et al., 1984; Wilson and Wilson, 1987; Reizer et al., 1994; Wilson and Ding, 2001). MelR is a member of the AraC/XylS transcription regulator family (Webster et al., 1989; Gallegos et al., 1997) and regulates the *melAB* operon in concert with melibiose (Webster et al., 1987) and cyclic AMP receptor (CRP) by oligomerising and binding to several binding sites in the region between the *melA* and *melR* coding sequences (Bourgerie et al., 1997; Belyaeva et al., 2000) in a very well defined and well studied order (Wade et al., 2001; Grainger et al., 2003; Kahramanoglou et al., 2006; Samarasinghe et al., 2008). In *melR*, we identified the same non-synonymous SNV at coding sequence position 118 to change a Threonine at codon 40 to a Proline in 16 independent replicates from the single drug treatments (DOX and ERY) and the C50 treatment (Table 4).

(ii) YjjG is nucleotidase from the haloacid dehalogenase (HAD)-like superfamily showing phosphatase activity on dTMP, dUMP, and UMP (Proudfoot et al., 2004; Kuznetsova et al., 2006). It can salvage a *thyA* mutant in its use of thymine as a nutrient (Weiss, 2007; Itsko and Schaaper, 2011). One of its major roles is probably as a house-cleaning enzyme, as it has been proven *in vivo* to protect against non-canonical pyrimidine derivatives by eventually preventing the incorporation of potentially mutagenic nucleotides into DNA (Titz et al., 2007). In *yjjG*, we found the same non-synonymous SNV at coding sequence position 138 to change an Asparagine at codon 46 to a Lysine in twelve independent replicates from the single drug treatments (DOX and ERY) and the C50 treatment (Table 4).

(iii) *yjjU* might encode a transcriptional regulator (Serres et al., 2001), is upregulated under mitomycin C induced DNA damage (Khil and Camerini-Otero, 2002) and has been inferred to be a lipid hydrolase by sequence homology (`http://www.uniprot.org/uniprot/P39407`). In *yjjU*, we identified the same non-synonymous SNV at coding sequence position 329 to change a Valine at codon 110 to a Glycine in six independent replicates from

the single drug treatments (DOX and ERY) and the C50 treatment (Table 4).

**Variants affected in combination treatments only (C50 and C100):** Three genes are specifically found to be variable in the C50 and C100 treatments, including *lon* (discussed above) and also *menC* and *rcnA*:

(i) *menC* (Sharma et al., 1993) is a gene encoding the O-succinylbenzoate synthase (OSBS) (Palmer et al., 1999; Thompson et al., 2000). This enzyme is part of the menoquinone biosynthesis pathway (Bentley and Meganathan, 1982), with the resulting menoquinone (or vitamin K2) being necessary for anaerobic growth (Newton et al., 1971). In one C50 and one C100_s replicate, the same variant was found. It is the signature of an IS*186* insertion into *menC* which has been reported before (Rozen and Lenski, 2000; Philippe et al., 2004). The resulting mutant phenotype S is suggested to have fitness advantages through possible cross-feeding and suppression of other phenotypes in stationary phase (Rozen and Lenski, 2000).

(ii) RcnA confers resistance to nickel and cobalt (Rodrigue et al., 2005), by being a nickel and cobalt efflux protein, involved in the homeostasis of these two compounds (Iwig et al., 2006; Koch et al., 2007). The repressor of *rcnA* expression, RcnR (Iwig et al., 2008; Iwig and Chivers, 2009), is in one protein family with FrmR (RcnR-FrmR-like_DUF156; for the *frmR* variant, see above) and both are in a superfamily with other transcriptional regulators, known to be involved in multidrug sensing (Liu et al., 2007). The respective SNV in *rcnA* is found in only one C50 replicate and results in an M75K substitution.

# 3 Supplementary Figures

**Supplementary Figure 1. Ancestry of MC4100 strains.** This is an overview of what we could reconstruct about the origins and the construction of MC4100 and its derivative strains – especially the MYMC4100 strain used in this study – from the cited sources.

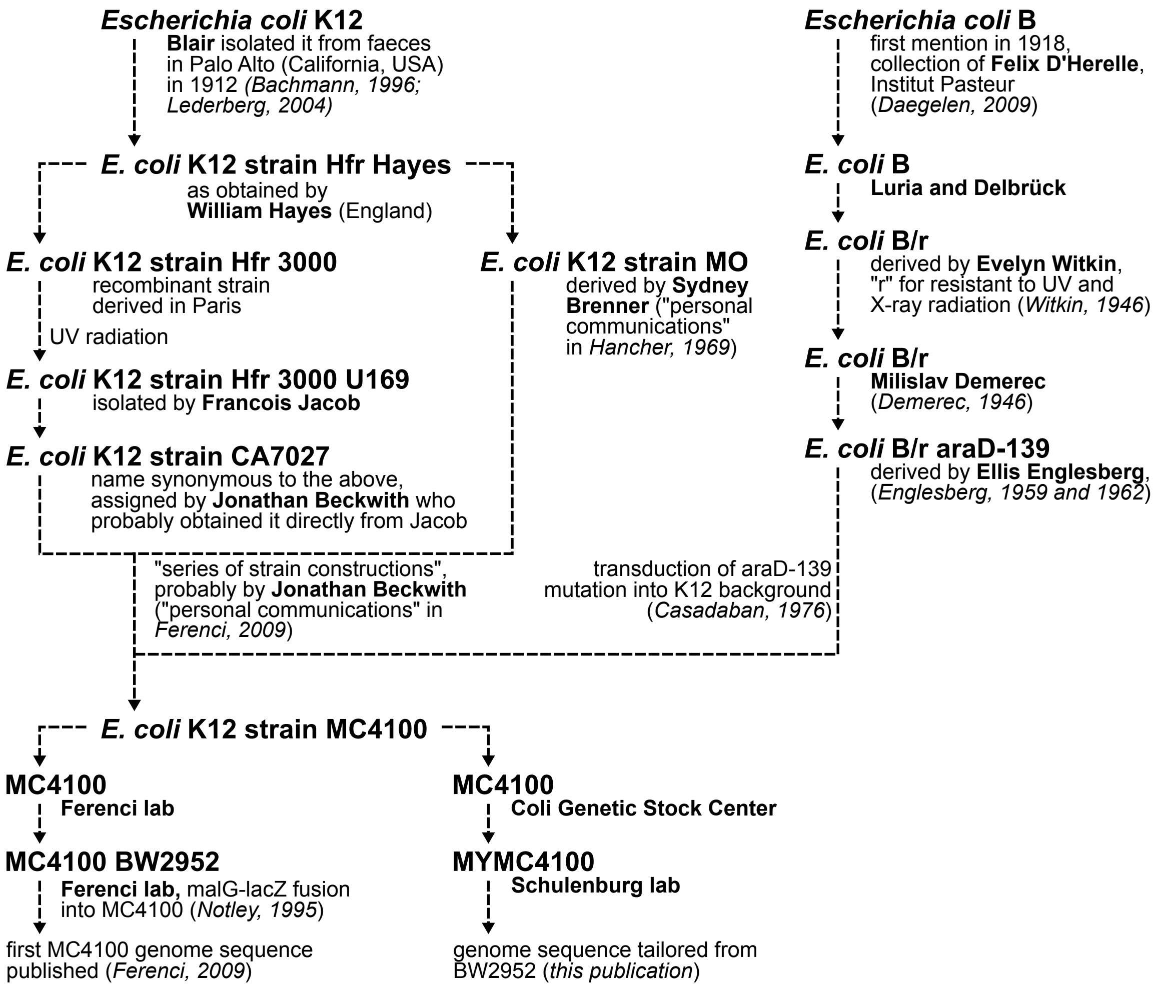

# 4 Supplementary Tables

**Supplementary Table 1.** Bioinformatics tools used for whole genome analysis

| Tool | Version | Reference | Used for |
|---|---|---|---|
| Jellyfish | 1.4.4 | Marçais and Kingsford (2011) | k-mer counting |
| Quake | 0.3.0 | Kelley et al. (2010) | error correction |
| SolexaQA | 1.10 | Cox et al. (2010) | quality trimming & filtering |
| bwa | 0.6.1-r104 | Li and Durbin (2009) | read mapping |
| SAMtools | 0.1.18 | Li et al. (2009) | mapping stats; sorting and indexing of sam-files |
| Picard | 1.69 | ?? | mapping stats; mark duplicates; extract unmapped reads |
| BEDtools | 2.16.2-7-gfd05a30 | Quinlan and Hall (2010) | computation of coverage |
| gap5 | 2.0.0b9 [a] | Bonfield and Whitwham (2010) | visual inspection of mappings |
| Pindel | 0.2.4s | Ye et al. (2009) | SV calling |
| CNVnator | 0.2.5 | Abyzov et al. (2011) | CNV calling |
| VarScan | 2.2.11 | Koboldt et al. (2012) | SNV and indel calling [b] |
| SNVer | 0.3.0 | Wei et al. (2011) | SNV calling [c] |
| VCFtools | 0.1.9 | Danecek et al. (2011) | merging of vcf-files |
| SnpEff | 3.0a | Cingolani et al. (2012) | annotation of variants |
| IS Finder | accessed 2012 | Siguier et al. (2006) | identify transposable elements |
| EcoCyc | 16.1 | Keseler et al. (2011) | review affected genes and literature |
| PolyPhen2 | 2.2.2 | Adzhubei et al. (2010) | predict effects of non-synonymous SNVs |
| Circos | 0.63-2 | Krzywinski et al. (2009) | circular coverage plot including highlights for variants |
| ggplot2 | 0.9.21 | Wickham (2009) | plotting average coverage of amplification region in R |

[a] Version number of the Staden package which contains gap5.
[b] Apart from SNVs, it can also detect indels and further SVs, e.g. CNVs. Nevertheless, it was only used for SNVs and indels here.
[c] Newer versions can also detect indels.

**Supplementary Table 2.** Comparison of SNVs detected and selected with the published reference BW2952 and our tailored reference MYMC4100

| | BW2952 | | | | MC4100 | | | |
|---|---|---|---|---|---|---|---|---|
| Gene | **SNVs**[a] | # DOX[b] | # ERY[b] | # C50[b] | **SNVs**[a] | # DOX[b] | # ERY[b] | # C50[b] |
| *acrB* | | | | | **1** | | 1 | |
| *acrR* | **1** | | 1 | | **2** | | 2 | |
| *agaS* | **1** | 1 | | | | | | |
| *ascF* | **1** | 1 | | | | | | |
| *clcB* | | | | | **1** | | 1 | |
| *dnaQ* | | | | | **1** | 1 | 1 | 2 |
| *eco* | **1** | 1 | | | | | | |
| *evgS* | **1** | | | 1 | | | | |
| *ftsP* | | | | | **3** | 2 | 2 | |
| *marR* | **7** | 5 | | | **4** | 4 | | |
| *mdaB* | **1** | 1 | | | **1** | 1 | | |
| *melR* | | | | | **1** | 4 | 5 | 7 |
| *mngB* | | | | | **1** | 2 | | 2 |
| *nudC* | | | | | **1** | | 2 | 3 |
| *qor* | | | | | **1** | 5 | 3 | 5 |
| *rcnA* | **1** | | | 1 | **1** | | | 1 |
| *recO* | | | | | **1** | 6 | 2 | 5 |
| *ycbZ* | **2** | | 3 | | **2** | | 3 | |
| *ydhW* | | | | | **1** | 2 | | |
| *yjjG* | | | | | **1** | 3 | 4 | 5 |
| *yjjU* | | | | | **1** | 1 | 1 | 4 |
| *yohF* | | | | | **1** | 1 | 2 | 2 |
| *ypfI* | | | | | **1** | | 2 | 1 |

[a] Number of unique SNVs across all treatments.
[b] Number of independent replicate populations affected by any of the SNVs in that gene and treatment.

**Supplementary Table 3.** p-Values from pairwise comparisons of the coverage means of all treatments as obtained by a Tukey HSD.
The average coverage is calculated as the average of positions 274,201 to 590,257 divided by the average of the rest of the genome. Significant differences are indicated by bold face, with the threshold defined as p ¡ 0.05.

| | noAB | DOX | ERY | C50 | C100_r_0 | C100_r_AB |
|---|---|---|---|---|---|---|
| DOX | 0.5556662 | | | | | |
| ERY | 0.1336412 | 0.9791058 | | | | |
| C50 | **0.0000002** | **0.0001808** | **0.0030250** | | | |
| C100_r_0 | **0.0077480** | 0.2809062 | 0.6997938 | 0.6055100 | | |
| C100_r_AB | **0.0000000** | **0.0000000** | **0.0000000** | **0.0057543** | **0.0002328** | |
| C100_s | 0.2297053 | 0.9992110 | 0.9994387 | **0.0003077** | 0.4356546 | **0.0000000** |

**Supplementary Table 4.** Detailed summary of variants sorted by the name of affected genes
Prevalence of the variants in independent replicates is given per gene and treatment.

| Gene | type[a] | position[b] | seq change[c] | effect[c,d] | DOX | ERY | C50 | C100_s | C100_r_0 | C100_r_AB | total |
|---|---|---|---|---|---|---|---|---|---|---|---|
| *acrA* | SNV[e] | 387,715[e] | c.-113A>C[e] | REG[e] | | | | 1[e] | | | 1[e] |
| *acrB* | SNV | 384,237 | c.2150C>A | p.R717L | | 1 | | | | | 1 |
| *acrR* | SNV[e] | 387,715[e] | c.-29A>C[e] | REG[e] | | | | 1[e] | | | 1[e] |
| | SNV | 387,756 | c.13A>G | p.T5A | | 1 | | | | | 1 |
| | SNV | 387,975 | c.232C>A | p.Q78K | | 1 | | 1 | | | 2 |
| *clcB* | SNV | 1,553,917 | c.74C>T | p.A25V | | 1 | | | | | 1 |
| *dnaQ* | SNV | 236,238 | c.173T>G | p.V58G | 1 | 1 | 2 | | | | 4 |
| *frmR* | indel | 281,996 | c.-407delG | REG? | | 1 | 1 | 1 | | 3 | 6 |
| *ftsP* | SNV | 3,620,438 | C.488T>G | p.V163G | 2 | 1 | | | | | 3 |
| | SNV[f]<br>SNV[f] | 3,621,122<br>3,621,123 | c.1172A>G[f]<br>c.1173C>G[f] | p.D391G<br>p.D391E | | 1[f]<br>1[f] | | | | | 1[f]<br>1[f] |
| *lon* | SV (IS*186*? cut-p?) | 360,770 | c.-101_100ins | REG | | | | | 1[g] | 1[g] | 2[g] |
| *marR* | indel | 1,507,691 | c.43_54del | DEL 4 codons | 1 | | | | | | 1 |
| | SNV | 1,507,728 | c.80G>C | p.R27P | | | | 1 | | | 1 |
| | indel | 1,507,829 | c.181delA | F | 2 | | | 1 | | | 3 |
| | indel | 1,507,829 | c.180_181insA | F | | | | 1 | | | 1 |
| | SV | 1,507,829 | c.181_242dup | F | 1 | | | | | | 1 |
| | indel | 1,507,833 | c.184_185insT | F | | | | 2 | | | 2 |
| | SNV | 1,507,856 | c.208G>A | p.A70T | 1 | | | | | | 1 |
| | SNV | 1,507,877 | c.229C>G | p.R77G | | | | 1 | | | 1 |
| | SNV | 1,507,881 | c.233T>G | p.L78R | | | | 2 | | | 2 |
| | SNV | 1,507,895 | c.247T>G | p.W83G | 1 | | | 1 | | | 2 |
| | SNV | 1,507,899 | c.251T>A | p.V84E | 1 | | | | | | 1 |
| | SV | 1,507,926 | c.278_349del | DEL 14 codons | | | | 1 | | | 1 |
| | indel | 1,507,933 | c.285delC | F | 1 | | | | | | 1 |
| | SNV | 1,507,959 | c.311G>A | p.G104D | 2 | | | | | | 2 |
| | indel | 1,508,076 | c.428delT | F | 1 | | | | | | 1 |

[a] Type of Variant. Abbreviations in this column: indel – small insertion/deletion, IS – insertion sequence, SNV – single nucleotide variant, SV – structural variant. [b] Nucleotide position in the tailored MYMC4100 reference genome. Variants affecting the same gene are sorted by position. [c] Nomenclature for the description of sequence variants as proposed by the Human Genome Variation Society: (Dunnen and Antonarakis, 2000); `http://www.hgvs.org/mutnomen/`. Abbreviations: c - coding sequence, p - protein sequence, del – deletion, dup – duplication, ins – insertion. [d] Abbr. in this column: D – disruption, F – frameshift, REG – affects regulatory sequences, SYN – synonymous SNVs. [e] This is the same SNV in the same sample, affecting the regulation of both *acrAB* and *acrR* through the AcrR binding site close to their promoter sequences. [f] These two SNVs in ftsP represent alternative alleles affecting the same codon / amino acid in the same sample. [g] These two samples are not independent. They represent the same experimental sample regrown with and without antibiotics for sequencing. [h] These variants occur in samples which also bear the *ycbZ* IS insertion (see below). [i] The recorded starting breakpoints of the *ycbZ* IS insertion event vary from this position to 920,454 between the four affected samples. [j] This SNV and indel represent different alleles of the same position, both present in the two same ERY samples.

**Supplementary Table 4.** Detailed summary of variants sorted by the name of affected genes
Prevalence of the variants in independent replicates is given per gene and treatment. **(continued)**

| Gene | type[a] | position[b] | DNA change[c] | effect[c,d] | DOX | ERY | C50 | C100_s | C100_r_0 | C100_r_AB | total |
|---|---|---|---|---|---|---|---|---|---|---|---|
| *mdaB* | SNV | 3,609,975 | c.116A>G | p.V39A | 1 | | | | | | 1 |
| *melR* | SNV | 4,227,357 | c.118T>G | p.T40P | 4 | 5 | 7 | | | | 16 |
| *menC* | SV (IS*186* cut-p) | 2,258,101 | c.135_136ins | D | | | 1 | 1 | | | 2 |
| *mngB* | SNV | 670,308 | c.348C>G | p.G116G, SYN REG? | 2 | | 2 | | | | 4 |
| *nudC* | SNV | 4,082,895 | c.37T>G | p.W13G | | 2 | 3 | | | | 5 |
| *qor* | SNV | 4,149,735 | c.453G>C | p.G151G, SYN REG? | 5 | 3 | 5 | | | | 13 |
| *rcnA* | SNV | 2,075,091 | c.224T>A | p.M75K | | | 1 | | | | 1 |
| *recO* | SNV | 2,584,278 | c.465G>C | p.G155G, SYN REG? | 6 | 2 | 5 | | | | 13 |
| *ycbZ* | indel | 918,916 | c.1574_1575insA | F | | 1 | | | | | 1 |
| | SV[h] | 918,958 | c.-39245_1533inv | D | | 1[h] | | | | | 1[h] |
| | SNV | 919,440 | c.1051G>A | p.Q351* | | 1 | | | | | 1 |
| | indel | 919,832 | c.659delT | F | | | | 1 | | | 1 |
| | indel[h] | 920,110 | c.381delT | F | | 1[h] | | | | | 1[h] |
| | SV (IS*3* copy-p) | 920,216[i] | c.274_275ins | D | | 4 | | | | | 4 |
| | SNV[j] | 920,225[j] | c.266C>G[j] | p.G98A[j] | | 2[j] | | | | | 2[j] |
| | indel[j] | 920,225[j] | c.266delC[j] | F[j] | | 2[j] | | | | | 2[j] |
| *ydhW* | SNV | 1,639,715 | c.539A>C | p.V180G | 2 | | | | | | 2 |
| *yjjG* | SNV | 4,494,378 | c.138C>A | p.N46K | 3 | 4 | 5 | | | | 12 |
| *yjjU* | SNV | 4,498,334 | c.329T>G | p.V110G | 1 | 1 | 4 | | | | 6 |
| *yohF* | SNV | 2,115,499 | c.723G>C | p.G241G, SYN REG? | 1 | 2 | 2 | | | | 5 |
| *ypfI* | SNV | 2,477,354 | c.779A>C | p.V260G | | 2 | 1 | | | | 3 |

[a] Type of Variant. Abbreviations in this column: indel – small insertion/deletion, IS – insertion sequence, SNV – single nucleotide variant, SV – structural variant. [b] Nucleotide position in the tailored MYMC4100 reference genome. Variants affecting the same gene are sorted by position. [c] Nomenclature for the description of sequence variants as proposed by the Human Genome Variation Society: Dunnen and Antonarakis (2000); `http://www.hgvs.org/mutnomen/`. Abbreviations: c - coding sequence, p - protein sequence, del – deletion, dup – duplication, ins – insertion. [d] Abbr. in this column: D – disruption, F – frameshift, REG – affects regulatory sequences, SYN – synonymous SNVs. [e] This is the same SNV in the same sample, affecting the regulation of both *acrAB* and *acrR* through the AcrR binding site close to their promoter sequences. [f] These two SNVs in ftsP represent alternative alleles affecting the same codon / amino acid in the same sample. [g] These two samples are not independent. They represent the same experimental sample regrown with and without antibiotics for sequencing. [h] These variants occur in samples which also bear the *ycbZ* IS insertion (see below). [i] The recorded starting breakpoints of the *ycbZ* IS insertion event vary from this position to 920,454 between the four affected samples. [j] This SNV and indel represent different alleles of the same position, both present in the two same ERY samples.

**Supplementary Table 5.** Overview of the four synonymous SNVs

| gene | position[a] | DNA change[b] | original mRNA context[c] | aa change |
|---|---|---|---|---|
| *mngB* | 670,308 | c.348C>G | `GCG TTT GG(C) GAG CCG ATG`[d] | p.G116G |
| *qor* | 4,149,735 | c.453G>C | `GCG GCT GG(C) GGC GTT GGC` | p.G151G |
| *recO* | 2,584,278 | c.465G>C | `GGT AGC GG(C) GAG CCG GTA`[d] | p.G155G |
| *yohF* | 2,115,499 | c.723G>C | `GTG GAT GG(C) GGC TTT ATG` | p.G241G |

[a] Position in the MYMC4100 sequence.
[b] DNA change as recorded by the SnpEff.
[c] The mRNA nucleotide C in parentheses was changed to a G in all four cases.
[d] Note the 9-nucleotide motif of similarity starting with the changed codon.

## References


Picard. version 1.69.

Abyzov A, Urban AE, Snyder M, Gerstein M. 2011, Jun. CNVnator: an approach to discover, genotype, and characterize typical and atypical CNVs from family and population genome sequencing. Genome Res. 21(6):974–984.

Accardi A, Miller C. 2004, Feb. Secondary active transport mediated by a prokaryotic homologue of ClC Cl- channels. Nature. 427(6977):803–807.

Adams MA, Jia Z. 2005, Mar. Structural and biochemical evidence for an enzymatic quinone redox cycle in *Escherichia coli*: identification of a novel quinol monooxygenase. J Biol Chem. 280(9):8358–8363.

Adzhubei IA, Schmidt S, Peshkin L, Ramensky VE, Gerasimova A, Bork P, Kondrashov AS, Sunyaev SR. 2010, Apr. A method and server for predicting damaging missense mutations. Nat Methods. 7(4):248–249.

Alekshun MN, Kim YS, Levy SB. 2000, Mar. Mutational analysis of MarR, the negative regulator of *marRAB* expression in *Escherichia coli*, suggests the presence of two regions required for DNA binding. Mol Microbiol. 35(6):1394–1404.

Alekshun MN, Levy SB. 1997, Oct. Regulation of chromosomally mediated multiple antibiotic resistance: the *mar* regulon. Antimicrob Agents Chemother. 41(10):2067–2075.

Anantharaman V, Aravind L. 2006, Feb. Diversification of catalytic activities and ligand interactions in the protein fold shared by the sugar isomerases, eIF2B, DeoR transcription factors, acyl-CoA transferases and methenyltetrahydrofolate synthetase. J Mol Biol. 356(3):823–842.

Bachmann BJ. 1996. *Escherichia coli* and *Salmonella typhimurium* : cellular and molecular biology, 2nd ed., Chapter Derivations and genotypes of some mutant derivatives of Escherichia coli. Washington, DC: American Society for Microbiology. p. 2460–2488.

Barbosa TM, Levy SB. 2000, Jun. Differential expression of over 60 chromosomal genes in *Escherichia coli* by constitutive expression of MarA. J Bacteriol. 182(12):3467–3474.

Belyaeva TA, Wade JT, Webster CL, Howard VJ, Thomas MS, Hyde EI, Busby SJ. 2000, Apr. Transcription activation at the *Escherichia coli melAB* promoter: the role of MelR and the cyclic AMP receptor protein. Mol Microbiol. 36(1):211–222.

Bentley R, Meganathan R. 1982, Sep. Biosynthesis of vitamin K (menaquinone) in bacteria. Microbiol Rev. 46(3):241–280.

Bessman MJ, Frick DN, O'Handley SF. 1996, Oct. The MutT proteins or "Nudix" hydrolases, a family of versatile, widely distributed, "housecleaning" enzymes. J Biol Chem. 271(41):25059–25062.

Bolton JL, Trush MA, Penning TM, Dryhurst G, Monks TJ. 2000, Mar. Role of quinones in toxicology. Chem Res Toxicol. 13(3):135–160.

Bonfield JK, Whitwham A. 2010, Jul. Gap5–editing the billion fragment sequence assembly. Bioinformatics. 26(14):1699–1703.

Bork JM, Cox MM, Inman RB. 2001, Dec. The RecOR proteins modulate RecA protein function at 5' ends of single-stranded DNA. EMBO J. 20(24):7313–7322.

Bourgerie SJ, Michán CM, Thomas MS, Busby SJ, Hyde EI. 1997, May. DNA binding and DNA bending by the MelR transcription activator protein from *Escherichia coli*. Nucleic Acids Res. 25(9):1685–1693.

Brinkkötter A, Klöss H, Alpert C, Lengeler JW. 2000, Jul. Pathways for the utilization of N-acetyl-galactosamine and galactosamine in *Escherichia coli*. Mol Microbiol. 37(1):125–135.

Burstein C, Kepes A. 1971, Jan. The alpha-galactosidase from *Escherichia coli* K12. Biochim Biophys Acta. 230(1):52–63.

Buxton RS, Albrechtsen H, Hammer-Jespersen K. 1977, Aug. Overlapping transcriptional units in the deo operon of *Escherichia coli* K-12. evidence from phage Mu-1 insertion mutants. J Mol Biol. 114(3):287–300.

Casadaban MJ. 1976, July. Transposition and fusion of the *lac* genes to selected promoters in *Escherichia coli* using bacteriophage lambda and Mu. Journal of Molecular Biology. 104(3):541–555.

Chatterjee PK, Sternberg NL. 1995, Sep. A general genetic approach in *Escherichia coli* for determining the mechanism(s) of action of tumoricidal agents: application to DMP 840, a tumoricidal agent. Proc Natl Acad Sci U S A. 92(19):8950–8954.

Cingolani P, Platts A, Wang LL, Coon M, Nguyen T, Wang L, Land SJ, Lu X, Ruden DM. 2012, Apr. A program for annotating and predicting the effects of single nucleotide polymorphisms, SnpEff: SNPs in the genome of *Drosophila melanogaster* strain w ( 1118) ; iso-2; iso-3. Fly (Austin). 6(2):80–92.

Cox MM. 2007. Regulation of bacterial RecA protein function. Crit Rev Biochem Mol Biol. 42(1):41–63.

Cox MP, Peterson DA, Biggs PJ. 2010. SolexaQA: At-a-glance quality assessment of Illumina second-generation sequencing data. BMC Bioinformatics. 11:485.

Daegelen P, Studier FW, Lenski RE, Cure S, Kim JF. 2009, December. Tracing ancestors and relatives of *Escherichia coli* B, and the derivation of B strains REL606 and BL21(DE3). Journal of Molecular Biology. 394(4):634–643.

Dandanell G, Norris K, Hammer K. 1991, Dec. Long-distance *deoR* regulation of gene expression in *Escherichia coli*. Ann N Y Acad Sci. 646:19–30.

Danecek P, Auton A, Abecasis G, Albers CA, Banks E, DePristo MA, Handsaker RE, Lunter G, Marth GT, Sherry ST, McVean G, Durbin R, Group GPA. 2011, Aug. The variant call format and VCFtools. Bioinformatics. 27(15):2156–2158.

Demerec M. 1946, Feb. Induced mutations and possible mechanisms of the transmission of heredity in *Escherichia coli*. Proc Natl Acad Sci U S A. 32(2):36–46.

DiFrancesco R, Bhatnagar SK, Brown A, Bessman MJ. 1984, May. The interaction of DNA polymerase III and the product of the *Escherichia coli* mutator gene, *mutD*. J Biol Chem. 259(9):5567–5573.

Dunnen JTd, Antonarakis SE. 2000, January. Mutation nomenclature extensions and suggestions to describe complex mutations: A discussion. Hum. Mutat. 15(1):7–12.

Edwards KJ, Thorn JM, Daniher JA, Dixon NE, Ollis DL. 1994, Jul. Crystallization and preliminary X-ray diffraction studies on a soluble *Escherichia coli* quinone oxidoreductase. J Mol Biol. 240(5):501–503.

Eicher T, Brandstätter L, Pos KM. 2009, Aug. Structural and functional aspects of the multidrug efflux pump AcrB. Biol Chem. 390(8):693–699.

Elkins CA, Nikaido H. 2002, December. Substrate specificity of the RND-type multidrug efflux pumps AcrB and AcrD of *Escherichia coli* is determined predominately by two large periplasmic loops. Journal of Bacteriology. 184(23):6490–6498.

Englesberg E, Anderson RL, Weinberg R, Lee N, Hoffee P, Huttenhauer G, Boyer H. 1962, Jul. L-arabinose-sensitive, L-ribulose 5-phosphate 4-epimerase-deficient mutants of *Escherichia coli*. J Bacteriol. 84:137–146.

Ferenci T, Zhou Z, Betteridge T, Ren Y, Liu Y, Feng L, Reeves PR, Wang L. 2009, Jun. Genomic sequencing reveals regulatory mutations and recombinational events in the widely used MC4100 lineage of *Escherichia coli* K-12. J Bacteriol. 191(12):4025–4029.

Frick DN, Bessman MJ. 1995, Jan. Cloning, purification, and properties of a novel NADH pyrophosphatase. evidence for a nucleotide pyrophosphatase catalytic domain in MutT-like enzymes. J Biol Chem. 270(4):1529–1534.

Gallegos MT, Schleif R, Bairoch A, Hofmann K, Ramos JL. 1997, Dec. AraC/XylS family of transcriptional regulators. Microbiol Mol Biol Rev. 61(4):393–410.

Gonzalez CF, Proudfoot M, Brown G, Korniyenko Y, Mori H, Savchenko AV, Yakunin AF. 2006, May. Molecular basis of formaldehyde detoxification. characterization of two S-formylglutathione hydrolases from *Escherichia coli*, FrmB and YeiG. J Biol Chem. 281(20):14514–14522.

Grainger DC, Belyaeva TA, Lee DJ, Hyde EI, Busby SJW. 2003. Binding of the *Escherichia coli* MelR protein to the *melAB* promoter: orientation of MelR subunits and investigation of MelR-DNA contacts. Molecular Microbiology. 48(2):335–348.

Gross J, Englesberg E. 1959, November. Determination of the order of mutational sites governing L-arabinose utilization in *Escherichia coli* Br by transduction with phage P1bt. Virology. 9(3):314–331.

Gutheil WG, Holmquist B, Vallee BL. 1992, Jan. Purification, characterization, and partial sequence of the glutathione-dependent formaldehyde dehydrogenase from *Escherichia coli*: a class III alcohol dehydrogenase. Biochemistry. 31(2):475–481.

Hanatani M, Yazyu H, Shiota-Niiya S, Moriyama Y, Kanazawa H, Futai M, Tsuchiya T. 1984, Feb. Physical and genetic characterization of the melibiose operon and identification of the gene products in *Escherichia coli*. J Biol Chem. 259(3):1807–1812.

Hancher CW, Phares EF, Novelli GD, Kelmers AD. 1969. Large scale production of transfer ribonucleic acids from *E. coli* K-12 MO7. Biotechnol. Bioeng. 11(6):1055–1070.

Hayashi M, Ohzeki H, Shimada H, Unemoto T. 1996, Feb. NADPH-specific quinone reductase is induced by 2-methylene-4-butyrolactone in *Escherichia coli*. Biochim Biophys Acta. 1273(2):165–170.

Heidrich C, Templin MF, Ursinus A, Merdanovic M, Berger J, Schwarz H, de Pedro MA, Höltje JV. 2001, Jul. Involvement of N-acetylmuramyl-L-alanine amidases in cell separation and antibiotic-induced autolysis of *Escherichia coli*. Mol Microbiol. 41(1):167–178.

Herring CD, Blattner FR. 2004, Oct. Global transcriptional effects of a suppressor tRNA and the inactivation of the regulator *frmR*. J Bacteriol. 186(20):6714–6720.

Higgins MK, Bokma E, Koronakis E, Hughes C, Koronakis V. 2004, Jul. Structure of the periplasmic component of a bacterial drug efflux pump. Proc Natl Acad Sci U S A. 101(27):9994–9999.

Ikeuchi Y, Kitahara K, Suzuki T. 2008, Aug. The RNA acetyltransferase driven by ATP hydrolysis synthesizes N4-acetylcytidine of tRNA anticodon. EMBO J. 27(16):2194–2203.

Inoue J, Honda M, Ikawa S, Shibata T, Mikawa T. 2008, Jan. The process of displacing the single-stranded DNA-binding protein from single-stranded DNA by RecO and RecR proteins. Nucleic Acids Res. 36(1):94–109.

Inoue J, Nagae T, Mishima M, Ito Y, Shibata T, Mikawa T. 2011, Feb. A mechanism for single-stranded DNA-binding protein (SSB) displacement from single-stranded DNA upon SSB-RecO interaction. J Biol Chem. 286(8):6720–6732.

Itsko M, Schaaper RM. 2011, Sep. The *dgt* gene of *Escherichia coli* facilitates thymine utilization in thymine-requiring strains. Mol Microbiol. 81(5):1221–1232.

Iwig JS, Chivers PT. 2009, Oct. DNA recognition and wrapping by *Escherichia coli* RcnR. J Mol Biol. 393(2):514–526.

Iwig JS, Leitch S, Herbst RW, Maroney MJ, Chivers PT. 2008, Jun. Ni(II) and Co(II) sensing by *Escherichia coli* RcnR. J Am Chem Soc. 130(24):7592–7606.

Iwig JS, Rowe JL, Chivers PT. 2006, Oct. Nickel homeostasis in *Escherichia coli* - the *rcnR-rcnA* efflux pathway and its linkage to NikR function. Mol Microbiol. 62(1):252–262.

Iyer R, Iverson TM, Accardi A, Miller C. 2002, Oct. A biological role for prokaryotic ClC chloride channels. Nature. 419(6908):715–718.

Kahramanoglou C, Webster CL, El-Robh MS, Belyaeva TA, Busby SJW. 2006, May. Mutational analysis of the *Escherichia coli melR* gene suggests a two-state concerted model to explain transcriptional activation and repression in the melibiose operon. J Bacteriol. 188(9):3199–3207.

Kantake N, Madiraju MVVM, Sugiyama T, Kowalczykowski SC. 2002, Nov. *Escherichia coli* RecO protein anneals ssDNA complexed with its cognate ssDNA-binding protein: A common step in genetic recombination. Proc Natl Acad Sci U S A. 99(24):15327–15332.

Kato J, Nishimura Y, Yamada M, Suzuki H, Hirota Y. 1988, Sep. Gene organization in the region containing a new gene involved in chromosome partition in *Escherichia coli*. J Bacteriol. 170(9):3967–3977.

Kelley DR, Schatz MC, Salzberg SL. 2010. Quake: quality-aware detection and correction of sequencing errors. Genome Biol. 11(11):R116.

Keseler IM, Collado-Vides J, Santos-Zavaleta A, Peralta-Gil M, Gama-Castro S, Muñiz-Rascado L, Bonavides-Martinez C, Paley S, Krummenacker M, Altman T, Kaipa P, Spaulding A, Pacheco J, Latendresse M, Fulcher C, Sarker M, Shearer AG, Mackie A, Paulsen I, Gunsalus RP, Karp PD. 2011, Jan. EcoCyc: a comprehensive database of *Escherichia coli* biology. Nucleic Acids Res. 39(Database issue):D583–D590.

Khil PP, Camerini-Otero RD. 2002, Apr. Over 1000 genes are involved in the DNA damage response of *Escherichia coli*. Mol Microbiol. 44(1):89–105.

Koboldt DC, Zhang Q, Larson DE, Shen D, McLellan MD, Lin L, Miller CA, Mardis ER, Ding L, Wilson RK. 2012, Mar. VarScan 2: somatic mutation and copy number alteration discovery in cancer by exome sequencing. Genome Res. 22(3):568–576.

Koch D, Nies DH, Grass G. 2007, Oct. The RcnRA (YohLM) system of *Escherichia coli*: a connection between nickel, cobalt and iron homeostasis. Biometals. 20(5):759–771.

Kolodner R, Fishel RA, Howard M. 1985, September. Genetic recombination of bacterial plasmid DNA: effect of RecF pathway mutations on plasmid recombination in *Escherichia coli*. Journal of Bacteriology. 163(3):1060–1066.

Krzywinski M, Schein J, Birol I, Connors J, Gascoyne R, Horsman D, Jones SJ, Marra MA. 2009, Sep. Circos: an information aesthetic for comparative genomics. Genome Res. 19(9):1639–1645.

Kuznetsova E, Proudfoot M, Gonzalez CF, Brown G, Omelchenko MV, Borozan I, Carmel L, Wolf YI, Mori H, Savchenko AV, Arrowsmith CH, Koonin EV, Edwards AM, Yakunin AF. 2006, Nov. Genome-wide analysis of substrate specificities of the *Escherichia coli* haloacid dehalogenase-like phosphatase family. J Biol Chem. 281(47):36149–36161.

Lederberg J. 2004. *E. coli* K12. Microbiol. Today. 31:116.

Li H, Durbin R. 2009, Jul. Fast and accurate short read alignment with Burrows-Wheeler transform. Bioinformatics. 25(14):1754–1760.

Li H, Handsaker B, Wysoker A, Fennell T, Ruan J, Homer N, Marth G, Abecasis G, Durbin R, Subgroup GPDP. 2009, August. The Sequence Alignment/Map format and SAMtools. Bioinformatics. 25(16):2078–2079.

Li M, Gu R, Su CC, Routh MD, Harris KC, Jewell ES, McDermott G, Yu EW. 2007, Nov. Crystal structure of the transcriptional regulator AcrR from *Escherichia coli*. J Mol Biol. 374(3):591–603.

Lilley PE, Stamford NP, Vasudevan SG, Dixon NE. 1993, Jul. The 92-min region of the *Escherichia coli* chromosome: location and cloning of the *ubiA* and *alr* genes. Gene. 129(1):9–16.

Lindgren PK, Karlsson A, Hughes D. 2003, Oct. Mutation rate and evolution of fluoroquinolone resistance in *Escherichia coli* isolates from patients with urinary tract infections. Antimicrob Agents Chemother. 47(10):3222–3232.

Liu T, Ramesh A, Ma Z, Ward SK, Zhang L, George GN, Talaat AM, Sacchettini JC, Giedroc DP. 2007, Jan. CsoR is a novel *Mycobacterium tuberculosis* copper-sensing transcriptional regulator. Nat Chem Biol. 3(1):60–68.

Luisi-DeLuca C, Kolodner R. 1994, February. Purification and characterization of the *Escherichia coli* RecO protein: Renaturation of complementary single-stranded DNA molecules catalyzed by the RecO protein. Journal of Molecular Biology. 236(1):124–138.

Ma D, Alberti M, Lynch C, Nikaido H, Hearst JE. 1996, Jan. The local repressor AcrR plays a modulating role in the regulation of *acrAB* genes of *Escherichia coli* by global stress signals. Mol Microbiol. 19(1):101–112.

Ma D, Cook DN, Alberti M, Pon NG, Nikaido H, Hearst JE. 1993, Oct. Molecular cloning and characterization of *acrA* and *acrE* genes of *Escherichia coli*. J Bacteriol. 175(19):6299–6313.

Ma D, Cook DN, Alberti M, Pon NG, Nikaido H, Hearst JE. 1995. Genes *acrA* and *acrB* encode a stress-induced efflux system of *Escherichia coli*. Molecular Microbiology. 16(1):45–55.

Maneewannakul K, Levy SB. 1996, Jul. Identification for *mar* mutants among quinolone-resistant clinical isolates of *Escherichia coli*. Antimicrob Agents Chemother. 40(7):1695–1698.

Marçais G, Kingsford C. 2011, Mar. A fast, lock-free approach for efficient parallel counting of occurrences of k-mers. Bioinformatics. 27(6):764–770.

McLennan AG. 2006, Jan. The Nudix hydrolase superfamily. Cell Mol Life Sci. 63(2):123–143.

Middlemiss JK, Poole K. 2004, March. Differential impact of MexB mutations on substrate selectivity of the MexAB-OprM multidrug efflux pump of *Pseudomonas aeruginosa*. Journal of Bacteriology. 186(5):1258–1269.

Mikolosko J, Bobyk K, Zgurskaya HI, Ghosh P. 2006, Mar. Conformational flexibility in the multidrug efflux system protein AcrA. Structure. 14(3):577–587.

Morrison PT, Lovett ST, Gilson LE, Kolodner R. 1989, July. Molecular analysis of the *Escherichia coli recO* gene. Journal of Bacteriology. 171(7):3641–3649.

Mortensen L, Dandanell G, Hammer K. 1989, Jan. Purification and characterization of the *deoR* repressor of *Escherichia coli*. EMBO J. 8(1):325–331.

Nagao Y, Nakada T, Imoto M, Shimamoto T, Sakai S, Tsuda M, Tsuchiya T. 1988. Purification and analysis of the structure of alpha-galactosidase from *Escherichia coli*. Biochemical and Biophysical Research Communications. 151(1):236 – 241.

Nakashima R, Sakurai K, Yamasaki S, Nishino K, Yamaguchi A. 2011, Dec. Structures of the multidrug exporter AcrB reveal a proximal multisite drug-binding pocket. Nature. 480(7378):565–569.

Nestorovich EM, Danelon C, Winterhalter M, Bezrukov SM. 2002, Jul. Designed to penetrate: time-resolved interaction of single antibiotic molecules with bacterial pores. Proc Natl Acad Sci U S A. 99(15):9789–9794.

Newton NA, Cox GB, Gibson F. 1971, Jul. The function of menaquinone (vitamin K 2 ) in *Escherichia coli* K-12. Biochim Biophys Acta. 244(1):155–166.

Nicoloff H, Andersson DI. 2013. Lon protease inactivation, or translocation of the lon gene, potentiate bacterial evolution to antibiotic resistance. Molecular Microbiology.:n/a–n/a.

Nicoloff H, Perreten V, Levy SB. 2007, Apr. Increased genome instability in *Escherichia coli lon* mutants: relation to emergence of multiple-antibiotic-resistant (Mar) mutants caused by insertion sequence elements and large tandem genomic amplifications. Antimicrob Agents Chemother. 51(4):1293–1303.

Nicoloff H, Perreten V, McMurry LM, Levy SB. 2006, Jun. Role for tandem duplication and *lon* protease in AcrAB-TolC- dependent multiple antibiotic resistance (Mar) in an *Escherichia coli* mutant without mutations in *marRAB* or *acrRAB*. J Bacteriol. 188(12):4413–4423.

Nishino K, Yamaguchi A. 2001, Oct. Analysis of a complete library of putative drug transporter genes in *Escherichia coli*. J Bacteriol. 183(20):5803–5812.

Notley L, Ferenci T. 1995. Differential expression of mal genes under cAMP and endogenous inducer control in nutrient-stressed *Escherichia coli*. Molecular Microbiology. 16(1):121–129.

O'Brien P. 1991. Molecular mechanisms of quinone cytotoxicity. Chemico-Biological Interactions. 80(1):1 – 41.

Palmer DR, Garrett JB, Sharma V, Meganathan R, Babbitt PC, Gerlt JA. 1999, Apr. Unexpected divergence of enzyme function and sequence: "N-acylamino acid racemase" is o-succinylbenzoate synthase. Biochemistry. 38(14):4252–4258.

Partridge JD, Browning DF, Xu M, Newnham LJ, Scott C, Roberts RE, Poole RK, Green J. 2008, Feb. Characterization of the *Escherichia coli* K-12 *ydhYVWXUT* operon: regulation by FNR, NarL and NarP. Microbiology. 154(Pt 2):608–618.

Philippe N, Alcaraz JP, Coursange E, Geiselmann J, Schneider D. 2004, May. Improvement of pCVD442, a suicide plasmid for gene allele exchange in bacteria. Plasmid. 51(3):246–255.

Plotkin JB, Kudla G. 2011, Jan. Synonymous but not the same: the causes and consequences of codon bias. Nat Rev Genet. 12(1):32–42.

Proudfoot M, Kuznetsova E, Brown G, Rao NN, Kitagawa M, Mori H, Savchenko A, Yakunin AF. 2004, Dec. General enzymatic screens identify three new nucleotidases in *Escherichia coli*. biochemical characterization of SurE, YfbR, and YjjG. J Biol Chem. 279(52):54687–54694.

Quinlan AR, Hall IM. 2010, Mar. BEDTools: a flexible suite of utilities for comparing genomic features. Bioinformatics. 26(6):841–842.

Reed JL, Vo TD, Schilling CH, Palsson BO. 2003. An expanded genome-scale model of *Escherichia coli* K-12 (iJR904 GSM/GPR). Genome Biol. 4(9):R54.

Reizer J, Reizer A, Saier MH. 1994, Jun. A functional superfamily of sodium/solute symporters. Biochim Biophys Acta. 1197(2):133–166.

Rodrigue A, Effantin G, Mandrand-Berthelot MA. 2005, Apr. Identification of *rcnA* (*yohM*), a nickel and cobalt resistance gene in *Escherichia coli*. J Bacteriol. 187(8):2912–2916.

Ross D. 2004, Oct. Quinone reductases multitasking in the metabolic world. Drug Metab Rev. 36(3-4):639–654.

Routh MD, Su CC, Zhang Q, Yu EW. 2009, May. Structures of AcrR and CmeR: insight into the mechanisms of transcriptional repression and multi-drug recognition in the TetR family of regulators. Biochim Biophys Acta. 1794(5):844–851.

Rozen, Lenski. 2000, Jan. Long-term experimental evolution in *Escherichia coli*. VIII. dynamics of a balanced polymorphism. Am Nat. 155(1):24–35.

SaiSree L, Reddy M, Gowrishankar J. 2001, Dec. IS*186* insertion at a hot spot in the *lon* promoter as a basis for Lon protease deficiency of *Escherichia coli* B: identification of a consensus target sequence for IS*186* transposition. J Bacteriol. 183(23):6943–6946.

Samaluru H, SaiSree L, Reddy M. 2007, Nov. Role of SufI (FtsP) in cell division of *Escherichia coli*: evidence for its involvement in stabilizing the assembly of the divisome. J Bacteriol. 189(22):8044–8052.

Samarasinghe S, El-Robh MS, Grainger DC, Zhang W, Soultanas P, Busby SJW. 2008, May. Autoregulation of the *Escherichia coli melR* promoter: repression involves four molecules of MelR. Nucleic Acids Res. 36(8):2667–2676.

Sampaio MM, Chevance F, Dippel R, Eppler T, Schlegel A, Boos W, Lu YJ, Rock CO. 2004, Feb. Phosphotransferase-mediated transport of the osmolyte 2-o-alpha-mannosyl-d-glycerate in *Escherichia coli* occurs by the product of the *mngA* (*hrsA*) gene and is regulated by the *mngR* (*farR*) gene product acting as repressor. J Biol Chem. 279(7):5537–5548.

Scheuermann R, Tam S, Burgers PM, Lu C, Echols H. 1983, Dec. Identification of the epsilon-subunit of *Escherichia coli* DNA polymerase III holoenzyme as the *dnaQ* gene product: a fidelity subunit for DNA replication. Proc Natl Acad Sci U S A. 80(23):7085–7089.

Schmitt R. 1968, Aug. Analysis of melibiose mutants deficient in alpha-galactosidase and thiomethylgalactoside permease II in *Escherichia coli* K-12. J Bacteriol. 96(2):462–471.

Sekine Y, Aihara K, Ohtsubo E. 1999, Nov. Linearization and transposition of circular molecules of insertion sequence IS*3*. J Mol Biol. 294(1):21–34.

Seoane AS, Levy SB. 1995, Jun. Characterization of MarR, the repressor of the multiple antibiotic resistance (*mar*) operon in *Escherichia coli*. J Bacteriol. 177(12):3414–3419.

Serres MH, Gopal S, Nahum LA, Liang P, Gaasterland T, Riley M. 2001. A functional update of the *Escherichia coli* K-12 genome. Genome Biol. 2(9):RESEARCH0035.

Shabalina SA, Spiridonov NA, Kashina A. 2013, Feb. Sounds of silence: synonymous nucleotides as a key to biological regulation and complexity. Nucleic Acids Res. 41(4):2073–2094.

Sharma V, Meganathan R, Hudspeth ME. 1993, Aug. Menaquinone (vitamin K2) biosynthesis: cloning, nucleotide sequence, and expression of the *menC* gene from *Escherichia coli*. J Bacteriol. 175(15):4917–4921.

Siguier P, Filée J, Chandler M. 2006, Oct. Insertion sequences in prokaryotic genomes. Curr Opin Microbiol. 9(5):526–531.

Siguier P, Varani A, Perochon J, Chandler M. 2012. Exploring bacterial insertion sequences with ISfinder: objectives, uses, and future developments. Methods Mol Biol. 859:91–103.

Stajich JE, Block D, Boulez K, Brenner SE, Chervitz SA, Dagdigian C, Fuellen G, Gilbert JGR, Korf I, Lapp H, Lehväslaiho H, Matsalla C, Mungall CJ, Osborne BI, Pocock MR, Schattner P, Senger M, Stein LD, Stupka E, Wilkinson MD, Birney E. 2002, Oct. The Bioperl toolkit: Perl modules for the life sciences. Genome Res. 12(10):1611–1618.

Su CC, Rutherford DJ, Yu EW. 2007, Sep. Characterization of the multidrug efflux regulator AcrR from *Escherichia coli*. Biochem Biophys Res Commun. 361(1):85–90.

Sulavik MC, Houseweart C, Cramer C, Jiwani N, Murgolo N, Greene J, DiDomenico B, Shaw KJ, Miller GH, Hare R, Shimer G. 2001, Apr. Antibiotic susceptibility profiles of *Escherichia coli* strains lacking multidrug efflux pump genes. Antimicrob Agents Chemother. 45(4):1126–1136.

Symmons MF, Bokma E, Koronakis E, Hughes C, Koronakis V. 2009, Apr. The assembled structure of a complete tripartite bacterial multidrug efflux pump. Proc Natl Acad Sci U S A. 106(17):7173–7178.

Takiff HE, Chen SM, Court DL. 1989, May. Genetic analysis of the *rnc* operon of *Escherichia coli*. Journal of Bacteriology. 171(5):2581–2590.

Thompson TB, Garrett JB, Taylor EA, Meganathan R, Gerlt JA, Rayment I. 2000, Sep. Evolution of enzymatic activity in the enolase superfamily: structure of o-succinylbenzoate synthase from *Escherichia coli* in complex with Mg2+ and o-succinylbenzoate. Biochemistry. 39(35):10662–10676.

Thorn JM, Barton JD, Dixon NE, Ollis DL, Edwards KJ. 1995, Jun. Crystal structure of *Escherichia coli* QOR quinone oxidoreductase complexed with NADPH. J Mol Biol. 249(4):785–799.

Titz B, Häuser R, Engelbrecher A, Uetz P. 2007, May. The *Escherichia coli* protein YjjG is a house-cleaning nucleotidase in vivo. FEMS Microbiol Lett. 270(1):49–57.

Umezu K, Chi NW, Kolodner RD. 1993, May. Biochemical interaction of the *Escherichia coli* RecF, RecO, and RecR proteins with RecA protein and single-stranded DNA binding protein. Proceedings of the National Academy of Sciences. 90(9):3875–3879.

Valentin-Hansen P, Højrup P, Short S. 1985, Aug. The primary structure of the DeoR repressor from *Escherichia coli* K-12. Nucleic Acids Res. 13(16):5927–5936.

Van Dyk TK, Ayers BL, Morgan RW, Larossa RA. 1998, February. Constricted flux through the branched-chain amino acid biosynthetic enzyme acetolactate synthase triggers elevated expression of genes regulated by *rpoS* and internal acidification. Journal of Bacteriology. 180(4):785–792.

Vargiu AV, Nikaido H. 2012, Dec. Multidrug binding properties of the AcrB efflux pump characterized by molecular dynamics simulations. Proc Natl Acad Sci U S A. 109(50):20637–20642.

Vasilyeva OV, Kolygo KB, Leonova YF, Potapenko NA, Ovchinnikova TV. 2002, Aug. Domain structure and ATP-induced conformational changes in *Escherichia coli* protease Lon revealed by limited proteolysis and autolysis. FEBS Lett. 526(1-3):66–70.

Wade JT, Belyaeva TA, Hyde EI, Busby SJ. 2001, Dec. A simple mechanism for co-dependence on two activators at an *Escherichia coli* promoter. EMBO J. 20(24):7160–7167.

Wang H, Dzink-Fox JL, Chen M, Levy SB. 2001, May. Genetic characterization of highly fluoroquinolone-resistant clinical *Escherichia coli* strains from China: role of *acrR* mutations. Antimicrob Agents Chemother. 45(5):1515–1521.

Webber MA, Piddock LJ. 2001, May. Absence of mutations in *marRAB* or *soxRS* in *acrB*-overexpressing fluoroquinolone-resistant clinical and veterinary isolates of *Escherichia coli*. Antimicrob Agents Chemother. 45(5):1550–1552.

Webster C, Gardner L, Busby S. 1989, Nov. The *Escherichia coli melR* gene encodes a DNA-binding protein with affinity for specific sequences located in the melibiose-operon regulatory region. Gene. 83(2):207–213.

Webster C, Kempsell K, Booth I, Busby S. 1987. Organisation of the regulatory region of the *Escherichia coli* melibiose operon. Gene. 59(2-3):253–263.

Wei Z, Wang W, Hu P, Lyon GJ, Hakonarson H. 2011, Oct. SNVer: a statistical tool for variant calling in analysis of pooled or individual next-generation sequencing data. Nucleic Acids Res. 39(19):e132.

Weiss B. 2007, Mar. YjjG, a dUMP phosphatase, is critical for thymine utilization by *Escherichia coli* K-12. J Bacteriol. 189(5):2186–2189.

Wickham H. 2009. ggplot2: elegant graphics for data analysis. Springer New York.

Wilson DM, Wilson TH. 1987, Nov. Cation specificity for sugar substrates of the melibiose carrier in *Escherichia coli*. Biochim Biophys Acta. 904(2):191–200.

Wilson TH, Ding PZ. 2001, May. Sodium-substrate cotransport in bacteria. Biochim Biophys Acta. 1505(1):121–130.

Witkin EM. 1946, Mar. Inherited differences in sensitivity to radiation in *Escherichia coli*. Proc Natl Acad Sci U S A. 32(3):59–68.

Wu TH, Clarke CH, Marinus MG. 1990, Mar. Specificity of *Escherichia coli mutD* and *mutL* mutator strains. Gene. 87(1):1–5.

Yazyu H, Shiota-Niiya S, Shimamoto T, Kanazawa H, Futai M, Tsuchiya T. 1984, Apr. Nucleotide sequence of the *melB* gene and characteristics of deduced amino acid sequence of the melibiose carrier in *Escherichia coli*. J Biol Chem. 259(7):4320–4326.

Ye K, Schulz MH, Long Q, Apweiler R, Ning Z. 2009, Nov. Pindel: a pattern growth approach to detect break points of large deletions and medium sized insertions from paired-end short reads. Bioinformatics. 25(21):2865–2871.

Yu B, Edstrom WC, Benach J, Hamuro Y, Weber PC, Gibney BR, Hunt JF. 2006, Feb. Crystal structures of catalytic complexes of the oxidative DNA/RNA repair enzyme AlkB. Nature. 439(7078):879–884.

Yu EW, Aires JR, McDermott G, Nikaido H. 2005, Oct. A periplasmic drug-binding site of the AcrB multidrug efflux pump: a crystallographic and site-directed mutagenesis study. J Bacteriol. 187(19):6804–6815.

Zgurskaya HI, Nikaido H. 1999, Jan. AcrA is a highly asymmetric protein capable of spanning the periplasm. J Mol Biol. 285(1):409–420.

Zgurskaya HI, Nikaido H. 2000, Aug. Cross-linked complex between oligomeric periplasmic lipoprotein AcrA and the inner-membrane-associated multidrug efflux pump AcrB from *Escherichia coli*. J Bacteriol. 182(15):4264–4267.
